# HycDemux: a hybrid unsupervised approach for accurate barcoded sample demultiplexing in nanopore sequencing

**DOI:** 10.1186/s13059-023-03053-1

**Published:** 2023-10-05

**Authors:** Renmin Han, Junhai Qi, Yang Xue, Xiujuan Sun, Fa Zhang, Xin Gao, Guojun Li

**Affiliations:** 1 Research Center for Mathematics and Interdisciplinary Sciences, Shandong University, Qingdao, 266237 China; 2 BioMap Research, California, USA; 3 High Performance Computer Research Center, Institute of Computing Technology, Chinese Academy of Sciences, Beijing, 100190 China; 4 School of Medical Technolgoy, Beijing Institute of Technology, Beijing, 100085 China; 5 King Abdullah University of Science and Technology (KAUST), Computational Bioscience Research Center (CBRC), Computer, Electrical and Mathematical Sciences and Engineering (CEMSE) Division, Thuwal, 23955 Saudi Arabia

**Keywords:** Nanopore sequencing, Demultiplexing, Clustering

## Abstract

DNA barcodes enable Oxford Nanopore sequencing to sequence multiple barcoded DNA samples on a single flow cell. DNA sequences with the same barcode need to be grouped together through demultiplexing. As the number of samples increases, accurate demultiplexing becomes difficult. We introduce HycDemux, which incorporates a GPU-parallelized hybrid clustering algorithm that uses nanopore signals and DNA sequences for accurate data clustering, alongside a voting-based module to finalize the demultiplexing results. Comprehensive experiments demonstrate that our approach outperforms unsupervised tools in short sequence fragment clustering and performs more robustly than current state-of-the-art demultiplexing tools for complex multi-sample sequencing data.

**Supplementary Information:**

The online version contains supplementary material available at 10.1186/s13059-023-03053-1.

## Background

A barcode is a very short nucleotide sequence attached at the 3′- or 5′- end of a DNA sequence to state where the sequence comes. By incorporating a unique barcode into the library of DNA molecules, multiple DNA libraries are able to be sequenced simultaneously [1]. Usually, short nucleotide sequence corresponds to a barcode or special coded segment within a long read whose length is short than 100 nucleotides. Clustering or classifying the reads into bins based on these short nucleic acid fragments is the first step in high-throughput sequencing techniques like multiple sample sequencing and single cell protocols [2, 3]. Specifically, the barcoding technique has recently been introduced to Oxford Nanopore devices to sequence multiple barcoded DNA samples on a single flow cell [4, 5].

Oxford Nanopore sequencing is a rapidly developing technology that enables ultra-long sequencing in real time at low-cost. The key innovation of nanopore sequencing is the direct measurement of the electrical current signal (denoted as the *raw signal*) when a single-strand DNA passes through the nanopore. These raw signals are transferred to nucleic acid bases by base-calling for further analysis [6–8]. The translation from raw current signals to reads may introduce significant base-calling errors. Specifically, considering a 40-nt barcode and a base-calling system with 10% error, the possibility that a sequenced barcode is completely correct is $0.9^{40}\approx 0.014$, which can badly hamper the downstream analyses [9]. Especially, because of the high base-calling error, the Unique Molecular Identifiers (UMI) technique, which is shorter nucleotide sequence added to sequencing libraries to identify PCR duplicates, is rarely used in Nanopore sequencing [10, 11].

A number of methods have been devised to group biological sequences that are related. In early 2001, a tool named CD-HIT [12] is proposed for the clustering of a large number of sequences, based on pairwise alignment and greedy strategy. Later, improved methods [13, 14] of CD-HIT are also devised to cope with the next-generation sequencing data. Inspired by CD-HIT, DNACLUST [15] is proposed for taxonomic profiling. Recently, the mean shift algorithm has also been introduced by MeShClust [16] to reduce the side effect of parameter dependency in the greedy strategy. On the other hand, alignment-free similarity measures [17–20] have been utilized in sequence clustering [21, 22], by mapping DNA sequences into feature vectors. Furthermore, clustering tools have also been devised for specified purpose, e.g., the Starcode [23] and Bartender [24]. However, all of these methods could only utilize the information of base-called reads in Nanopore sequencing.

On the contrary, the raw current signal contains much more information compared with the base-called reads. In practice, the frequency of the electrical current measurements is 7$\sim$9 times higher than the passing speed of the DNA sequence, which makes the raw current signal to contain $\sim$ 8 $\times$ redundant information than the base-called read. Except for signal-level polishing [25], efforts have been made to utilize raw signal for targeted sequencing [8, 26, 27], variant identification [28, 29], and methylation detection [30–32]. Recently, the raw current signal has also been utilized in ONT barcode demultiplexing and achieved good results, by training a deep neural network as barcode’s raw signal classifier [33, 34]. Here, the demultiplexing is carried out as a supervised machine learning task with the classifier trained under a large human-labeled dataset. However, a problem with the supervised-learning-based classification is that the performance of these methods heavily depends on the training dataset.

**Fig. 1:**
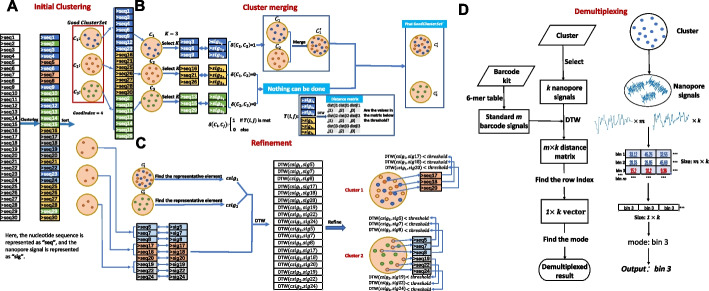
An example of clustering 30 sequences using our hybrid clustering algorithm (**A**, **B**, and **C**) and the subsequent demultiplexing mechanism based on the clustering results (**D**). **A** We perform initial clustering on 30 sequences, resulting in 6 clusters. If a cluster contains a number of sequences greater than the *GoodIndex*, it is considered a good cluster. In this case, clusters $C_1$, $C_2$, and $C_3$ are considered good clusters. **B** We attempt to merge the good clusters by selecting *k* signals from each cluster. If the distance values in the corresponding $k \times k$ dynamic time warping (DTW) distance matrix are all smaller than a given threshold, we merge two clusters. In this case, $C_1$ and $C_2$ are merged into a single cluster. **C** We attempt to add sequences that are not in good clusters to the existing good clusters after the cluster merging is completed. We select a representative sequence from each good cluster and calculate the DTW distance between the representative sequence and the sequences not in the good cluster. If the distance value is less than a given threshold, we add the sequence to the corresponding good cluster. **D** We demultiplex the cluster by selecting *k* signals within the cluster and converting all known barcode sequences into standard nanopore signals based on the official 6-mer table of Oxford Nanopore Sequencing Company. We then calculate the DTW distance matrix between these *k* signals and the standard nanopore signals, and find the row label corresponding to the minimum value of each column to obtain a one-dimensional row label matrix. Finally, we compute the mode of the row-labeled matrix and use it as the final demultiplexed result

In this paper, we first demonstrate that, for Nanopore sequencing, signal-similarity (dynamic time warping distance)-based clustering performs much better than the base-space clustering in various criteria (Additional file 1: S1), though the computation of pair-wise signal similarity is computationally expensive (Additional file 1: S2). Consequently, we propose HycDemux, which integrates a GPU-parallelized hybrid clustering algorithm and a voting module for the accurate clustering of short sequence fragments and demultiplexing of barcoded samples in nanopore sequencing. Our approach utilizes both the base-called nucleic base information and the raw current signal, in which the nucleotides are used to generate initial clustering and representation sequences, while the raw signals are used for cluster merging and refinement (Fig. 1 (A, B, and C) gives an example). A checking mechanism is built to make sure that the good sequences are reserved and correctly grouped. We compared our hybrid clustering algorithm with traditional DNA clustering tools and found that our algorithm provides more complete clustering results ($>95.5$%) while ensuring high homogeneity ($>99.7$%). The completeness of our method is about 30% higher than traditional clustering tools, providing a strong guarantee for subsequent demultiplexing. The results of high completeness and high homogeneity imply that our hybrid clustering algorithm can find out the barcodes that can be successfully generated in the dataset, which has potential significance for the design of barcodes. To transform clustering results into the final demultiplexed results, we designed a module based on a voting mechanism (Fig. 1D). Comprehensive experiments show that combining our hybrid clustering algorithm with this module leads to more accurate and robust demultiplexing results. When applied to multi-sample sequencing data generated by Nanopore’s official barcode suites, our method performs comparably to the state-of-the-art method. In particular, we evaluated our algorithm on datasets with different sequencing error rates, regarding different nanopore sequencing kits [35–38]. For complex sequencing data (number of barcodes = 350, sequencing error 10% $\sim$ 15%), we achieve a demultiplexing accuracy of above 90% for each barcode, which is about 30% higher than state-of-the-art method and 15% higher than state-of-the-art method on the low error rate datasets. It is important to note that the field of sequencing technology is continually advancing, leading to enhanced sequencing accuracy. This improvement suggests that our algorithms will yield even better results in future studies. In addition, our algorithm incorporates a GPU-based parallel mechanism, which allows for the demultiplexing of 3.5 GB (gigabytes) of data (nanopore signals + base-called DNA sequences) in approximately 1 min.

## Results

We have developed a comprehensive pipeline to extract pseudo-barcode regions from raw sequencing data. All of the extracted data is then utilized for subsequent clustering and demultiplexing. In regard to the extracted pseudo-barcode regions, HycDemux integrates an unsupervised hybrid approach to achieve accurate and efficient clustering, in which the nucleotides-based greedy algorithm is utilized to obtain initial clusters (Initial clustering), and the raw signal information is measured to guide the continuously optimization and refinement of clustering results (cluster merging and cluster refinement). GPU acceleration based on CUDA technique is utilized in our hybrid clustering (GPU-accelerated DTW). On the other hand, HycDemux integrates a module that uses a voting mechanism to determine the final demultiplexing result. This module selects *n* representatives (5 by default) for each cluster and calculates the DTW distance matrix between these representatives and the standard barcode signal. By identifying the row index of the minimum value in each column of the distance matrix, the module determines which barcode the sequence belongs to. As a result, *n* demultiplexing results are obtained, and the barcode with the highest frequency in the result determines the demultiplexing outcome of this cluster. The detailed implementation is explicated in the “Materials and methods” section.

We derive the results of demultiplexing by the results of hybrid clustering, implying that the results of clustering directly affect the results of demultiplexing. In this section, we first evaluate the performance of HycDemux’s hybrid clustering algorithm, and the experimental results show that it can generate high-quality clusters, which provides a strong guarantee for subsequent demultiplexing. Afterwards, we show that a voting-based demultiplexing module can derive demultiplexed results with high accuracy from clustered results.

### Evaluation of hybrid clustering algorithm

We demultiplex sequences based on the results of hybrid clustering algorithm, which poses a performance challenge for clustering. Here, we mainly evaluate the performance of clustering from two aspects: one is homogeneity, and the other is completeness. High homogeneity means that the sequences in each cluster obtained by clustering have the same barcode. However, there is an extreme case where each cluster contains only one sequence and the homogeneity is 100%. Therefore, the clustering results also need to be evaluated through completeness. High completeness means that there is a relatively small difference between the number of clusters obtained in the end and the actual number of barcodes.

Starting from the clustering results, the demultiplexing results are deduced. The main advantage is that under the clustering results with high homogeneity, the demultiplexing result of a sequence are determined by some sequences in the cluster where it is located. This makes the result of demultiplexing more robust. On the other hand, we point out that clustering does not affect the efficiency of demultiplexing. Given *n* sequences, assuming that these *n* sequences carry *m* different barcodes, in theory, the final demultiplexing result can be obtained by completing $n \times m$ alignments. Clustering *n* sequences, assuming that $n_1$ clusters are finally obtained, and then pick *k* representative elements in each cluster, and the *k* representative elements determine the result of demultiplexing, which requires $n_1 \times k \times m$ alignments, when the clustering result has high completeness, $n_1 \times k$ will be much smaller than *n*, which means that the efficiency of demultiplexing will be greatly improved. At the same time, clustering results with high homogeneity will lead to highly accurate demultiplexing results.

We conducted experiments to demonstrate the hybrid clustering algorithm’s ability to produce high homogeneity and completeness results. The experimental process and analysis are presented in detail below.

#### Simulated datasets

A set of synthetic datasets with different configurations are generated. Here, we first generate a set of random barcodes and then produce a number of these barcodes’ copies as well as their raw signals by DeepSimulator. The configuration of synthetic dataset includes the following three points:

- The length of barcode (nucleotide sequence length)
- The number of clusters within a dataset
- The number of sequences for the whole dataset

Finally, we construct 12 simulated datasets. The details of these datasets are provided in Additional file 1: S3.

#### Real-world datasets

The real-world dataset came from eight R9.4 flow cells and six R9.5 flow cells, all sequenced with the EXP-NBD103 barcoding kit. We conducted a random selection of 130,000 sequences from the dataset provided by [33] and proceeded to calculate the edit distance between the barcode area in each sequence and the standard barcode area. If the edit distance exceeded 10, we labeled the sequence as “fuzzy,” indicating uncertainty regarding the presence of barcodes in these particular sequences. Ultimately, we constructed barcode labels for 120,947 sequences, forming a dataset known as the amplicon library. In amplicon library, we use *Edlib* [39] to locate the fixed region of the barcode and segment the barcode read and use *Semi-Global Dynamic Time* Warping [40] to extract the corresponding raw signal of these barcodes.

All the aforementioned datasets primarily consist of two main components. The first component comprises sequence fragments that represent the barcodes, while the second component consists of the nanopore signals that correspond to the barcode sequences.

#### Run scripts

DNACLSUT fails to cope with dataset large than 10,000 sequences. Therefore, here we mainly compare our hybrid clustering with CD-HIT, UCLUST, MeShClust. The command line options for these three clustering tools are listed as follows:

1. CD-HIT: ./cd-hit-est -i infile.fasta -o outfile.fasta -c indentity
2. MeShClust: ./meshclust infile.fasta –id identity -output outfile.fasta
3. UCLSUT: ./usearch -cluster_fast infile.fasta -id identity -clusters output

All the experiments were run on an Ubuntu 18.04 system with Intel(R) Core(TM) i9-10980XE (18 cores), 128 Gb memory, and an NVIDIA RTX3080 card.

#### Evaluation on synthetic datasets

Six synthetic datasets with different barcode lengths, numbers of clusters, and data sizes are selected to demonstrate the performance of HycDemux. Table 1 describes the details of the six selected datasets.

**Table 1:** Summaries of the details about the selected synthetic datasets

Dataset	Barcode length	Number of clusters	Data size
$S_{1}$	45nt	20	50000
$S_{2}$	70nt	20	50000
$S_{3}$	95nt	20	50000
$S_{4}$	45nt	50	50000
$S_{5}$	70nt	50	50000
$S_{6}$	95nt	50	50000

Table 2 summarizes the experimental results of different clustering methods on these synthetic datasets, where the indexes AMI, FMI, ACC, HOMO, COMP, and runtime are adapted for the performance evaluation. Detailed information on all evaluation metrics can be found in the Additional file 1: S1.

**Table 2:** Performance comparison of the different clustering methods on the dataset$S_1 \sim S_6$. Here, Identity is the parameter of clustering tools, AMI is the abbreviation of adjusted mutual information, FMI is the abbreviation of Fowlkes-Mallows Index, ACC is the abbreviation of accuracy, HOMO is the abbreviation of homogeneity, and COMP is the abbreviation of completeness

Dataset	Tool	Identity (%)	AMI (%)	FMI (%)	ACC (%)	HOMO (%)	COMP (%)	Time (min:sec)
S_1_	MeShClust	80.0	58.17	34.51	42.16	64.46	54.57	1:07.81
		85.0	58.31	36.65	44.31	67.53	53.91	1:28.03
		90.0	62.69	40.18	36.35	86.60	51.69	0:58.17
		95.0	56.66	33.69	24.86	94.59	45.62	1:00.59
	CD-HIT	80.0	77.63	66.28	57.32	100.00	64.61	0:01.03
		85.0	70.51	59.47	49.78	100.00	57.06	0:01.60
		90.0	58.73	45.50	36.92	100.00	47.41	0:02.67
		95.0	41.30	26.92	18.71	100.00	37.89	0:04.00
	UCLUST	80.0	61.74	47.37	37.97	100.00	49.06	0:00.59
		85.0	54.53	38.50	28.34	100.00	44.09	0:00.80
		90.0	44.19	27.02	17.64	100.00	38.64	0:01.09
		95.0	32.67	18.10	11.48	100.00	34.33	0:01.44
	HycDemux	-	97.73	97.40	96.75	99.99	95.59	0:10.95
S_2_	MeShClust	80.0	67.10	50.96	57.66	70.45	64.98	0:55.28
		85.0	67.63	52.12	52.67	83.91	58.61	1:12.78
		90.0	61.96	50.08	41.15	92.98	50.83	1:00.49
		95.0	59.67	49.57	44.31	96.26	49.03	1:56.98
	CD-HIT	80.0	81.89	71.30	65.57	100.00	70.02	0:01.40
		85.0	73.60	60.10	52.22	100.00	60.09	0:02.36
		90.0	60.24	42.76	33.59	100.00	48.08	0:04.63
		95.0	40.52	24.03	16.17	100.00	37.37	0:07.25
	UCLUST	80.0	63.74	45.82	35.77	100.00	50.27	0:00.96
		85.0	53.73	32.44	22.27	100.00	43.06	0:01.35
		90.0	40.88	20.52	13.63	100.00	36.89	0:01.91
		95.0	26.29	12.46	7.34	100.00	32.38	0:02.44
	HycDemux	-	99.89	99.91	99.91	100.00	95.59	0:13.12
S_3_	MeShClust	80.0	85.06	83.40	86.24	93.44	79.06	1:33.12
		85.0	79.99	81.27	79.19	96.50	70.48	1:58.35
		90.0	66.37	65.43	61.96	94.12	56.85	2:01.84
		95.0	57.65	58.07	45.25	99.47	48.83	1:07.26
	CD-HIT	80.0	88.70	83.32	78.95	100.00	79.99	0:01.65
		85.0	81.22	74.83	67.45	100.00	69.48	0:03.20
		90.0	67.86	60.14	52.22	100.00	55.24	0:06.92
		95.0	44.53	30.26	21.45	100.00	39.50	0:04.00
	UCLUST	80.0	68.75	54.50	44.48	100.00	55.08	0:01.29
		85.0	58.44	44.18	32.51	100.00	46.91	0:01.79
		90.0	44.36	26.50	18.00	100.00	38.76	0:02.46
		95.0	26.93	14.09	8.60	100.00	32.67	0:01.44
	HycDemux	-	97.78	96.90	95.39	100.00	95.67	0:19.34
S_4_	MeShClust	80.0	54.00	24.31	32.06	65.57	50.39	0:47.93
		85.0	49.58	17.13	27.23	59.03	47.54	0:57.78
		90.0	55.31	28.55	23.48	87.42	49.55	1:01.96
		95.0	50.60	26.03	19.31	92.11	47.40	1:04.56
	CD-HIT	80.0	78.29	59.75	50.20	99.99	67.34	0:01.28
		85.0	69.95	50.26	40.07	100.00	59.80	0:01.89
		90.0	56.29	36.47	26.12	100.00	51.10	0:03.31
		95.0	35.86	20.55	12.87	100.00	43.17	0:04.77
	UCLUST	80.0	61.21	39.56	29.19	100.00	53.59	0:00.79
		85.0	53.42	32.61	22.30	100.00	49.51	0:01.03
		90.0	40.48	21.84	13.65	100.00	44.40	0:01.46
		95.0	27.05	14.30	8.36	100.00	40.78	0:01.85
	HycDemux	-	98.42	97.03	96.28	99.73	97.18	0:19.96
S_5_	MeShClust	80.0	68.39	47.68	52.91	82.86	62.65	1:03.37
		85.0	62.40	36.39	46.49	82.04	57.61	1:43.73
		90.0	54.90	24.28	33.06	73.30	52.13	1:55.66
		95.0	53.89	24.53	31.02	82.26	51.23	1:06.23
	CD-HIT	80.0	85.39	71.10	64.08	100.00	75.94	0:01.66
		85.0	77.87	60.19	51.56	100.00	67.23	0:02.76
		90.0	65.62	48.89	39.54	100.00	57.13	0:04.93
		95.0	45.10	28.76	20.16	100.00	46.40	0:07.29
	UCLUST	80.0	70.56	55.00	43.23	100.00	60.60	0:01.13
		85.0	60.71	44.25	32.51	100.00	53.79	0:01.56
		90.0	46.99	30.48	20.19	100.00	47.03	0:02.12
		95.0	30.91	18.12	10.80	100.00	41.81	0:02.50
	HycDemux	-	98.77	97.43	95.90	99.99	97.61	0:29.50
S_6_	MeShClust	80.0	87.66	79.22	83.36	93.81	83.40	0:49.57
		85.0	83.44	76.77	77.05	95.24	76.69	1:01.72
		90.0	76.70	71.47	68.35	96.18	68.72	1:03.75
		95.0	70.43	64.56	56.08	98.07	62.35	1:05.74
	CD-HIT	80.0	92.46	86.29	82.23	100.00	86.44	0:01.58
		85.0	86.30	77.45	72.41	100.00	77.51	0:02.80
		90.0	74.23	61.01	52.30	100.00	64.25	0:05.54
		95.0	52.12	32.98	24.42	100.00	49.34	0:09.23
	UCLUST	80.0	78.99	67.27	57.71	100.00	68.73	0:01.27
		85.0	69.14	54.09	44.34	100.00	59.75	0:01.73
		90.0	53.36	34.91	25.64	100.00	49.81	0:02.39
		95.0	31.74	15.71	9.68	100.00	41.86	0:03.07
	HycDemux	-	99.40	99.11	98.93	100.00	98.82	0:33.77

Judging from the experimental results, it can be found that CD-HIT and UCLUST are able to guarantee high homogeneity (HOMO index $=100\%$) under various situations. The high homogeneity is reasonable because CD-HIT and UCLUST are designed to maintain the consistency of the elements within a cluster. UCLUST is the fastest and CD-HIT is the second fastest in clustering speed, because of the utility of non-alignment technique. When the sequence length is very short, MeShClust behaves the poorest within the four clustering methods, as shown in Table 2.

Drown from the six synthetic datasets, we can make the following key conclusions:

- CD-HIT and UCLUST are the fastest and able to guarantee high homogeneity (HOMO more than 98%) of the results.
- For barcodes with short sequence length ($S_1$ and $S_4$), the clustering performance of MeShClust is very poor. With the increase of barcode length, the clustering performance of MeShClust is significantly improved.
- The clustering results of $S_1$ to $S_3$ and $S_4$ to $S_6$ demonstrate that the performance of clustering tools depends on the length of barcode sequence, and longer sequence results in better clustering result.
- HycDemux outperforms other tools significantly in terms of clustering performance, provided that speed is ensured. HycDemux achieves a completeness of over 95%, which is more than 30% higher than other tools, while ensuring high homogeneity (> 99.7%). This ensures accuracy in subsequent demultiplexing and improves overall efficiency.
- The speed of HycDemux is affected by the number of clusters residing in the dataset, as the result comparison of $S_1$ and $S_4$, $S_2$ and $S_5$, and $S_3$ and $S_6$. This is caused by the fact that the number of clusters determines the number of DTW distance that should be calculated in the cluster merging phase.

In general, due to the base-calling error, the traditional clustering tools such as CD-HIT, UCLUST, and MeShClust could not get good clustering results in the analysis of short nanopore reads. In particular, the clustering completeness of these tools is poor. For a dataset containing 50,000 sequences and 20 clusters, these tools may produce results with more than 1000 clusters. However, HycDemux significantly improves completeness while ensuring clustering speed, resulting in fewer than 100 clusters. The initial clustering of our method guarantees the extremely high homogeneity within the clusters, and the cluster merging and refinement guarantee the high accuracy and completeness of the clustering, which greatly reduces the influence of base-calling error. The results show that our method produces very good clustering results, and the results produced by traditional clustering tools cannot compete with us. This greatly benefits subsequent demultiplexing processes. More benchmarking results are provided in Additional file 1: Table S10 $\sim$ Table S15.

#### Performance analysis of different stages

As introduced in previous sections, the hybrid clustering algorithm is composed by three stages, i.e., initial clustering, cluster merge, and cluster refinement. In this section, we analyze the detailed contributions of different stages in hybrid clustering and their time cost.

**Fig. 2:**
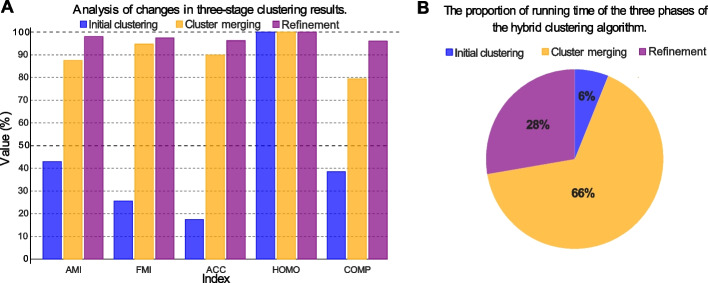
**A** The clustering result of hybrid clustering at different stages on dataset $S_{3}$. The results after initial clustering, cluster merging and cluster refinement is labeled by red, purple and green, respectively. **B** Pie chart of different stages’ runtime percentage of the algorithm on $S_{3}$

First, we analyze the change of cluster accuracy of these different stages. As shown in Fig. 2A, after the initial clustering, the clustering result is not so good. With the completion of the merging phase, the clustering performance has been greatly improved. After the refinement phase, the clustering result is further improved. The change of index values in Fig. 2A clearly show the effectiveness of the three-stage solution in our hybrid clustering algorithm. Especially, the signal based cluster merging and refinement contributes a lot to the accuracy improvement in clustering result.

Then, we analyze the time cost of these different stages. Figure 2B shows the runtime of the three stages for the clustering of $S_3$ in pie chart. As shown in Fig. 2B, the hybrid clustering algorithm spends the most time in the cluster merging stage. This is reasonable, since a large number of DTW distance comparisons are computed in cluster merging.

#### Speedup of GPU-accelerated DTW

As described in the previous section, numerous DTW distance are calculated in our algorithm, but we still achieve relatively small time cost. Here, we would like to show the benefits of GPU acceleration in DTW distance calculation.

**Fig. 3:**
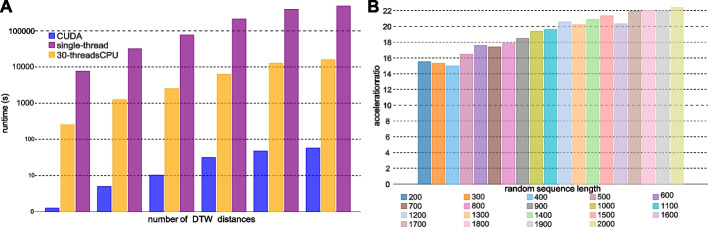
**A** The time comparison between different acceleration strategy with different data size. **B** Acceleration ratio for random sequences with different lengths, where the acceleration ratio = (runtime of single-thread DTW)/(runtime of single-thread CUDA)

In order to show the overall acceleration effect, we generate a large amount of time series as test data, whose details are shown in Table 3. We compare the CUDA implementation of DTW with CPU single-threaded method and CPU multi-threaded method. The DTW method realized by CUDA is equivalent to the original DTW in the mathematical model, which can guarantee its correctness. The CPU single-threaded method is a naive DTW algorithm. In the CPU multi-threaded approach, each CPU thread is responsible for calculating the DTW distance of a pair of time series, while the single CPU thread still uses the traditional method for calculation. Figure 3A clearly shows the time spent by different approaches in logarithmic scale. As shown in Fig. 3A, the CUDA accelerated DTW is at least three orders of magnitude faster than the traditional single-threaded DTW, and two orders of magnitude faster than the 30-threaded DTW.

**Table 3:** Three different kinds of time series used for the comparison of different DTW’s implementation, where all the time series are with a length of 1300. “Random” means time series of random walk. “Simulator” means current signals generated by DeepSimulator. “Amplicon library” means the real data downloaded from [33]. Here, we divide the data into two groups, and the time series within each group will compare with each other. For example, in the first random dataset,$200\times 1000$means that the first group has 200 time series, the second group has 1000 time series, and the number of DTW comparison is$200 \times 1000 = 200000$

Source	Size	DTW numbers
Random	200$\times$1000	200000
Random	1000$\times$1000	1000000
Simulator	200$\times$10000	2000000
Random	200$\times$30000	6000000
Random	1000$\times$10000	10000000
Amplicon library	100$\times$120947	12094700

In addition, we evaluate the DTW acceleration ratio of different lengths by simulated nanopore signals generated from DeepSimulator, where the length of the current signal is approximately 8 times of that of the corresponding DNA template. Figure 3B shows the change of acceleration ratio with different sequence lengths, where the acceleration ratio for single DTW calculation ranges from 14$\times$ to 22$\times$, increasing with the lengthening of sequences. Furthermore, as introduced in previous section, a block-wise acceleration strategy is proposed to fully utilize the advantage of GPU blocks, which enables the launch of million threads of DTW calculation simultaneously. In Supplementary Table S1, we have shown that it takes about 1100 min to calculate the DTW distance matrix of $2000\times 2000$. As a comparison, by applying the CUDA acceleration strategy, the time cost of the DTW distance matrix calculation can be reduced to 4 s.

#### Runtime analysis of the hybrid clustering algorithm

As discussed in previous section, the hybrid clustering algorithm consists of three stages and the calculation of DTW distance is GPU-accelerated. Here, we would like to further analyze the overall time complexity of the hybrid clustering algorithm.

**Fig. 4:**
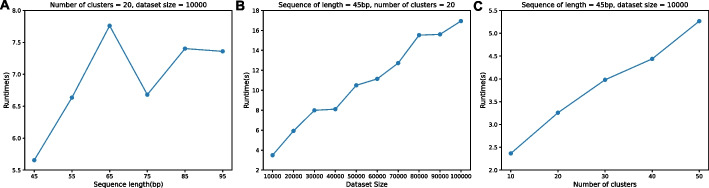
The runtime of hybrid clustering on **A** a dataset with 10,000 sequences and 20 clusters when the length of the sequences changes, **B** a dataset with about 45 nt long sequences and 20 clusters when the size changes, and **C** a dataset with long sequences when the number of clusters changes

We simulated a number of datasets by DeepSimulator to test the time cost of our algorithm under different sequence lengths, dataset sizes, and numbers of clusters, whose results are summarized in Fig. 4. Figure 4A shows that the runtime of our algorithm is not simply correlated with the sequence length. When the sequence length is 75 nt, the total time cost of our algorithm is smaller than the one with 75 nt sequence. In addition, the time costs for datasets with 75 nt, 85 nt, and 95 nt sequence length are almost the same. In fact, the accuracy of initial clustering could benefit from longer sequence, which shortens the runtime of further cluster merging and refinement. Figure 4B and C shows that the runtime of our algorithm linearly increases with the increment of dataset size and number of clusters, which ensures an acceptable time cost even when the size of dataset is relatively large. In practice, our method can complete barcode clustering efficiently.

#### Evaluation on real-world dataset

The real-world dataset is downloaded from [33], composed of 12 classes of nanopore barcodes, with $\sim$ 40 base pairs for each barcode. For the real-world barcode sequences, the first 8 positions of nucleobase and the last 8 positions of nucleobase are same to each other. Thus, the base-calling error may translate two identical nanopore barcode into different nucleobase reads, which greatly hampers the correctness of clustering and classification.

Table 4 summarizes the experimental results of different clustering methods on the real-world dataset. As shown in Table 4, the performance of MeShClust on the real-world dataset is very poor at all identities, failing to guarantee even the homogeneity of clustering. At each identity, the performance of the CD-HIT is slightly better than that of UCLUST, while UCLUST can always guarantee higher HOMO. The performance of HycDemux on the real-world dataset is much better than that of the other classic clustering tools, with 99.47% homogeneity and 83.22% completeness. In terms of clustering efficiency, UCLUST and CDHIT still maintain a clear advantage, while the hybrid clustering algorithm can also complete the clustering of about one hundred thousand sequences within very short time. By fully utilizing the raw signal information, HycDemux can cope with the challenge of base-calling error well, outperforming the classic ‘base-space’ clustering tools. Especially, our algorithm has finished the clustering within 15 s which is also very efficient.

**Table 4:** Performance comparison of the different clustering methods on the real-world dataset

Tool	Identity (%)	AMI (%)	FMI (%)	ACC (%)	HOMO (%)	COMP (%)	Time (min:sec)
MeShClust	80.0	0.20	30.37	15.93	0.32	19.02	0:51.35
CD-HIT	80.0	77.86	71.68	64.68	96.31	65.57	0:02.36
UCLUST	80.0	56.86	35.70	20.96	99.96	41.24	0:02.2
MeShClust	85.0	4.63	29.53	17.13	3.14	25.76	1:31.95
CD-HIT	85.0	70.21	56.74	50.29	99.77	54.71	0:03.14
UCLUST	85.0	50.08	27.07	16.35	99.97	36.12	0:01.73
MeShClust	90.0	27.06	23.52	23.02	24.44	34.50	3:02.26
CD-HIT	90.0	56.88	34.81	27.64	99.96	41.60	0:07.89
UCLUST	90.0	40.92	18.21	12.35	99.98	30.93	0:02.79
MeShClust	95.0	26.54	21.75	18.98	29.42	30.36	7:31.98
CD-HIT	95.0	39.71	18.30	13.56	99.98	30.72	0:21.57
UCLUST	95.0	31.10	13.39	9.63	99.99	27.32	0:04.32
HycDemux	—-	90.60	91.55	88.19	99.47	83.22	0:13.45

### Evaluation of the demultiplexing in HycDemux

Previous studies have demonstrated that hybrid clustering algorithm can deliver clustering results with high homogeneity and completeness. In this context, we will elaborate on how our hybrid clustering algorithm, coupled with a voting mechanism-based demultiplexing module, can attain demultiplexing results with high accuracy. We compare our method with the state-of-the-art demultiplexing tool, Guppy, and provide experiment details below.

#### Simulated multi-sample sequencing data

**Fig. 5:**
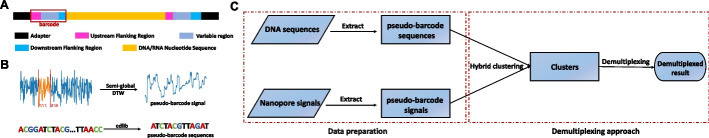
Data preparation for evaluating demultiplexing and the pipeline of HycDemux. **A** DNA sequence after multi-sample sequencing library construction. **B** Extract “barcode” sequences (nanopore signals) within sequences (nanopore signals) using a fast heuristic scheme (see the "Materials and methods" section). **C** Complete pipeline of HycDemux for demultiplexing consists of several steps. First, the data preparation pipeline completes the necessary preprocessing of the data. Next, the hybrid clustering algorithm (Fig. 1A, B, C) performs the data clustering process. Finally, the demultiplexing module (Fig. 1D) completes the final step of demultiplexing

We obtained whole genome sequences for 17 *Enterotoxigenic Escherichia coli *strains [41], 45 *historical Shigella *strains [42], and 67 *Shiga toxin-producing Escherichia coli *strains [43] to construct multi-sample sequencing datasets. We constructed multiple multi-sample sequencing libraries by randomly interrupting genome sequences based on the sequencing length distribution of Oxford Nanopore Sequencing Technology (ONT). Figure 5A illustrates the resulting DNA sequence after library construction. We used a total of 11 multi-sample sequencing datasets (Table 5) to evaluate our algorithm; all datasets were mixed with an additional 1000 sequences that either lacked or had incomplete barcode regions (with a missing ratio greater than 0.6). These sequences were classified as negative samples and their correct barcode label should be “unclassified.” In contrast, sequences containing the complete barcode region were categorized as positive samples. In addition, D1$\sim$D7 carried higher sequencing errors (10$\sim$15%), and DB4$\sim$DB7 carried lower sequencing errors (2$\sim$ 5%).

In these datasets, D1$\sim$D3 integrate the official nanopore barcode. In order to evaluate the robustness of the demultiplexing method and the performance of demultiplexing a large number of non-ONT barcodes [44, 45], we increased the number of barcodes and generated simulated multi-sample sequencing data (D4$\sim$D7 and DB4$\sim$DB7), these barcodes were randomly generated, and a certain edit distance was guaranteed (edit distance = 14.5 ± 1.8). The barcode consist of three primary components: upstream flanking region, variable region, and downstream flanking region (Fig. 5A). While the barcode lengths vary among these datasets, the variable regions remain consistent at 24 nt in length.

For the EXP-NBD104 kit (D1), both upstream flanking region and downstream flanking region are 8 nt long, resulting in a total barcode length of 40 nt. In the case of the SQK-16S024 kit (D2), upstream flanking region spans 15 nt, downstream flanking region covers 20 nt, and the barcode itself is 59 nt in length. Finally, the EXP-PBC096 kit (D3) features a upstream flanking region of 7 nt, a downstream flanking region of 29 nt, and an overall barcode length of 60 nt. For barcodes in D4$\sim$D7 and DB4$\sim$DB7, both upstream flanking region and downstream flanking region are 8nt long, resulting in a total barcode length of 40 nt.

**Table 5:** All datasets used to evaluate demultiplexing performance. “GB” is an abbreviation for gigabytes

Dataset	No.of reads	Barcoding kit	Size
D1	13, 000	Nanopore, EXP-NBD104, 12 barcodes	3.54 GB
D2	25, 000	Nanopore, SQK-16S024, 24 barcodes	6.73 GB
D3	97, 000	Nanopore, EXP-PBC096, 96 barcodes	26.76 GB
D4/DB4	200, 956	Random, 200 barcodes	52.63 GB
D5/DB5	250, 946	Random, 250 barcodes	65.73 GB
D6/DB6	300, 937	Random, 300 barcodes	78.83 GB
D7/DB7	350, 920	Random, 350 barcodes	91.92 GB

#### Extract data for demultiplexing

We obtained the barcode sequences (signals) from the multi-sample sequencing dataset (Fig. 5B). However, as errors may occur during the extraction process, we refer to the barcode sequence (signal) as a pseudo-barcode sequence (signal). These extracted pseudo-barcode sequences (signals) are utilized in the hybrid clustering and demultiplexing that follow (Fig. 5C).

#### Evaluation index

Our analysis encompasses the demultiplexing accuracy of each individual barcode, employing two evaluation metrics: average accuracy and minimum accuracy. Now, we explain the concepts of average accuracy and minimum accuracy using an example. Consider a scenario with 10 sequences labeled as $read_1, read_2, ..., read_{10}$. The correct barcode labels for these sequences are 1, 1, 1, 1, 1, 2, 2, 2, 2, 2. Here, the label “1” (or “2”) indicates that the sequence carries the 1st (or 2nd) barcode. We want to assess the accuracy rates for these two barcodes. Assuming that the barcode labels obtained by the demultiplexing algorithm for the 10 sequences are 1, 2, 1, 1, 2, 1, 1, 2, 1, 2, we can calculate the accuracy rates. The accuracy rate for the first barcode is 3/5, indicating that 3 out of 5 sequences labeled as barcode 1 are correct. Similarly, the accuracy rate for the second barcode is 2/5, as 2 out of 5 sequences labeled as barcode 2 are correct. In this case, average accuracy is calculated as (3/5 + 2/5)/2 = 0.5, and minimum accuracy is $min(3/5,2/5)=0.4$. It is important to note that when dealing with a large number of barcodes, it is possible to have a demultiplexing result with a high average accuracy but a low minimum accuracy. This means that the algorithm performs well on the majority of barcodes but may be ineffective for certain barcodes. Therefore, solely relying on the average accuracy might not provide a comprehensive evaluation of the demultiplexing effectiveness. Supplementary Fig. S1 further demonstrates the importance of minimum accuracy. By including the minimum accuracy, we can better assess the performance of the demultiplexing algorithm.

In addition, we utilize the recall rate as a measure of the model’s performance in correctly identifying positive samples. The formula for calculating the recall rate is: Recall = TP / (TP + FN). Here, TP represents true positive examples (the number of samples correctly predicted as positive by the model), and FN represents false negative examples (the number of samples that are actually positive but are incorrectly predicted as negative by the model).

#### Performance on all datasets

We conducted extensive experiments on all datasets to showcase the effectiveness of HycDemux, and the main experimental results are presented in Tables 6 and 7. As shown in Table 6, both HycDemux and Guppy achieve nearly perfect accuracy on datasets D1 $\sim$ D3, which have a limited number of carefully designed ONT barcodes (not exceeding 96). As the number of barcodes increases in D4$\sim$D7, Guppy’s average accuracy remains around 0.95 but its minimum accuracy drops below 0.7, indicating that Guppy fails to demultiplex sequences associated with certain barcodes. In contrast, HycDemux maintains a stable performance with a minimum accuracy above 0.9. In terms of recall, HycDemux outperforms Guppy by approximately 3%. Additionally, we observed that HycDemux exhibits fewer instances of “unclassified” labels compared to Guppy, and it aligns more closely with the ground truth value of 1000. This indicates that our demultiplexing algorithm excels at accurately assigning the correct barcode label to each sequence.

**Table 6:** Performance of HycDemux and Guppy (GPU version) on D1$\sim$D7 with a 10$\sim$15% sequencing error rate

Dataset	Minimum accuracy		Average accuracy		Recall		Unclassified		Run time (m:s)	
	Guppy	HycDemux	Guppy	HycDemux	Guppy	HycDemux	Guppy	HycDemux	Guppy	HycDemux
D1	0.9940	0.9820	0.9990	0.9905	0.9990	0.9899	1012	1109	0:08	0:17
D2	0.9880	0.9840	0.9972	0.9918	0.9971	0.9919	1066	1146	0:24	0:54
D3	0.9740	0.9530	0.9961	0.9892	0.9960	0.9894	1330	1604	4:31	7:18
D4	0.6170	0.9620	0.9554	0.9898	0.9552	0.9897	9732	2616	5:15	12:07
D5	0.4150	0.9430	0.9519	0.9872	0.9518	0.9871	12,794	3208	8:01	19:54
D6	0.4150	0.9450	0.9516	0.9895	0.9514	0.9894	15,253	3443	11:19	25:15
D7	0.4150	0.9260	0.9489	0.9902	0.9488	0.9901	18,623	3565	15:19	33:49

The randomly selected barcodes in D4$\sim$D7 may contain basecalling errors, which could impede Guppy’s demultiplexing accuracy based on DNA sequences. Additionally, as the number of barcodes increases, distinguishing between some barcodes becomes increasingly challenging, thereby making demultiplexing more difficult. HycDemux utilizes both DNA sequence and nanopore signal information to achieve highly homogeneous clustering results and avoid basecalling errors. The voting mechanism is used to obtain demultiplexing results, which prevents abnormal sequences from affecting the accuracy of demultiplexing.

As shown in Table 7, with the improvement of sequencing error rate, both HycDemux and Guppy showed improved demultiplexing accuracy, which is expected as sequencing error rates are generally inversely related to algorithm’s accuracy. In terms of average accuracy and recall, HycDemux demonstrated an advantage of approximately 2% over Guppy. In addition, it is worth noting that even with these improvements, the minimum accuracy of Guppy remains below 0.8, and HycDemux outperforms Guppy by $\sim$15%. This shows that under the current state-of-the-art sequencing accuracy, Guppy still cannot successfully demultiplex some samples, while HycDemux guarantees a demultiplexing accuracy above 0.9.

Compared to Guppy, HycDemux is slightly less efficient in terms of speed but the running time remains at the same order of magnitude, due to the fact that HycDemux involves a lot of DTW distance calculations. However, it is important to note that HycDemux still achieves a high level of demultiplexing efficiency. In our test environment, the extraction efficiency of barcode data is around $\sim$255 reads/s. Based on this estimate, the time required to complete the demultiplexing of D1 is 17 + 47 = 64 s, which means that our method can complete the demultiplexing of 3.5 G data in $\sim$ 1 min.

**Table 7:** Performance of HycDemux and Guppy (GPU version) on DB4$\sim$DB7 with a 2$\sim$5% sequencing error rate

Dataset	Minimum accuracy		Average accuracy		Recall		Unclassified		Run time (m:s)	
	Guppy	HycDemux	Guppy	HycDemux	Guppy	HycDemux	Guppy	HycDemux	Guppy	HycDemux
DB4	0.8240	0.9310	0.9704	0.9931	0.9702	0.9932	6840	2013	6:15	6:28
DB5	0.7860	0.9440	0.9715	0.9920	0.9713	0.9921	7967	2645	7:53	8:25
DB6	0.7860	0.9170	0.9718	0.9921	0.9717	0.9922	9177	2879	11:09	12:30
DB7	0.7920	0.9410	0.9723	0.9894	0.9722	0.9893	10,325	3867	15:01	19:11

## Discussion

We perform demultiplexing based on the clustering results, which offers a significant advantage. Clustering, particularly in clusters with high homogeneity, determines the demultiplexing outcome of a sequence based on other sequences within the same cluster. This characteristic enhances the robustness of the demultiplexing process, as it ensures that sequences within a cluster contribute to the determination of the demultiplexed result. Nanopore sequencing produces two types of data, i.e., the raw current signals and base-called reads. For barcode sequence clustering, the first consideration is what kind of data should be used for clustering. We found that the direct use of raw signal information combined with the DTW algorithm can produce good clustering performance, but the time cost is high (Additional file 1: S2). Using the read information combined with existing clustering tools is fast but cannot produce good clustering completeness. The hybrid clustering algorithm makes use of these two types of data for clustering. In the initial clustering stage, the read information is used to generate the initial clustering results, and in the cluster merging and refinement stages, the raw signal information is used to continuously refine the initial clustering results. From the experimental results of simulated datasets and real datasets, the clustering accuracy of the hybrid clustering algorithm is obviously better than that of various classic clustering tools. Additionally, we have integrated a GPU-based module into our algorithm, specifically designed for computing the DTW distance matrix between time series. This module proves highly efficiency of GPU powered clustering, when dealing with time series datasets. The utilization of GPU for distance computation and clustering has been crucial, as evidenced by our experiments. By harnessing the power of GPUs, we have effectively applied certain algorithms that are slow but accurate, such as DTW distance computation with a complexity of $n^2$, to big data analysis. This has ensured both the accuracy and efficiency of the analysis process, and has the potential to inspire future work in this area.

Through extensive experiments, we made an interesting observation regarding clustering tools and their clustering accuracy. While some clustering tools may not achieve high clustering accuracy, we found that certain tools utilizing greedy strategies, such as CDHIT, can ensure near-perfect homogeneity. This discovery has led to the emergence of a new clustering concept: employing a greedy strategy to rapidly obtain highly homogeneous clusters and subsequently merging these clusters in a careful manner to continually improve clustering accuracy. By employing a suitable merging strategy for these initial clusters, we can achieve clustering results with significantly higher accuracy. Additionally, the complexity of clustering is substantially reduced when starting from these initial clusters, as compared to the original sequence set. This strategy can be seamlessly applied to DNA sequence clustering problems once an appropriate cluster merging scheme is established.

Our demultiplexing module is designed based on the hybrid clustering algorithm, which yields highly homogeneous and integrated clustering results. By employing a voting mechanism for demultiplexing each cluster, HycDemux achieves more accurate and stable demultiplexing results.

There is still room for improvement in HycDemux. Currently, we employ a heuristic scheme to extract the pseudo-barcode sequence (signal) by relying on the relationship between the length of the nanopore signal and the length of the DNA sequence. While experimental results have shown its effectiveness, there are cases where we cannot guarantee that the extracted pseudo-barcode signal contains sufficient useful information. To address this concern and prevent it from affecting the final demultiplexing results, we have adopted a simple designed DTW distance threshold (as described in the "Extract barcode information from raw data" section). In future research, we will focus on enhancing our algorithm in this aspect.

In recent years, significant advancements have been made in ONT Direct RNA sequencing. This approach eliminates the need for reverse transcription of RNA into cDNA, thereby mitigating potential issues associated with introducing errors or losing information during transcription. However, it is important to note that individual sequencing of RNA molecules often yields data with a relatively high error rate [46, 47]. On the other hand, the combination of RNA molecules and barcodes also enables multi-sample sequencing [34]. Through experiments, we can see that our demultiplexing algorithm can successfully complete the demultiplexing of multiple samples on datasets with a high error rate, which implies that our algorithm can be applied to the demultiplexing of RNA samples. This is also the focus of our future work.

Furthermore, barcoding is not only applicable to the multi-sample sequencing but also finds significant utility in the realm of single-cell RNA sequencing. By employing the 10X method in conjunction with ONT sequencing, RNA isoforms can be quantified at the individual cell level. The combination of ONT sequencing and the 10X method generates vast amounts of data, encompassing thousands of barcodes. These barcodes originate from a “white list” consisting of millions of barcodes. In downstream analysis, accurately identifying the barcodes within the sequences is the crucial initial step, as sequences with the same barcode are presumed to originate from the same cell. In response to this specific challenge, we aim to develop a more adaptive algorithm building upon our current work.

## Conclusion

This paper presents an approach named HycDemux for barcoded sample demultiplexing in nanopore sequencing.

HycDemux initially obtains highly homogeneous clusters using the hybrid clustering algorithm and then employs a voting mechanism module to perform demultiplexing. HycDemux delivers stable performance, particularly when there is a large number of samples. It ensures a demultiplexing accuracy of $>0.9$ per sample, which is approximately 0.3 higher than the accuracy of the state-of-the-art method on the high error rate datasets and 0.15 higher than the state-of-the-art method on the low error rate datasets. On the other hand, experiments on datasets with high error rates imply that HycDemux can be applied to direct RNA sequencing problems, especially RNA demultiplexing of multiple samples. Specifically, the introducing of GPU-acceleration significantly reduce the execution time of signal similarity comparison, which makes the processing of a huge number of data possible. In addition, the experimental evaluation of GPU-based DTW calculation demonstrates the efficient utilization of GPUs in clustering analysis. This approach ensures both efficiency and accuracy in the measurement process, offering valuable insights and reference for related research endeavors.

## Materials and methods

### Overview

**Fig. 6:**
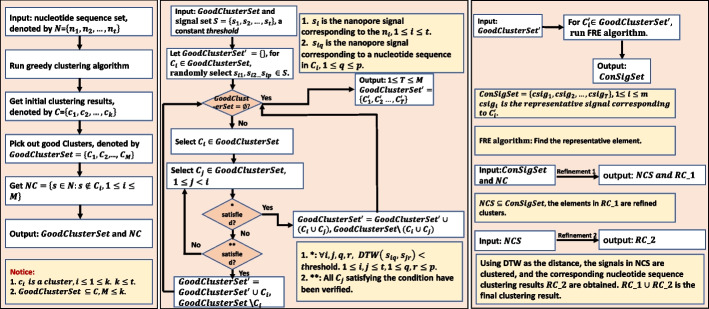
The workflow of hybrid clustering integrated in HycDemux. First, a greedy clustering algorithm is used to obtain initial clustering based on the nucleotide information. Then, we try to merge the initial clusters with good homogeneity by the information of raw signals, in which the threshold of signal-to-signal DTW distance is determined with a partial sampling method. For each merged cluster, a corresponding representative signal is produced. Finally, based on these representative signals, a signal-similarity based classification strategy is designed to assign the unclassified sequences to the already known clusters, to further refine the clustering result

We have designed a heuristic scheme to extract pseudo-barcode sequences (signals) in raw data for subsequent clustering and demultiplexing. For these pseudo-barcode sequences (signals), we developed an unsupervised hybrid approach, in which the nucleobase-based greedy algorithm is utilized to obtain initial clusters, and the raw signal information is measured to guide the continuously optimization and refinement of clustering results. Figure 6 shows the detailed workflow of hybrid clustering.

Given the nanopore sequences, we first utilize the nucleotide information for *initial clustering* to generate clusters with high homogeneity (identity $\geqslant$ 95%), whose process is based on a greedy clustering strategy and very quick. Then, we select some sequences in the clusters for *threshold determination* for subsequent cluster merging and refinement. Finally, we make the *cluster merging and refinement* by calculating the DTW distance between the raw signals and each cluster’s representative signals, with *GPU-accelerated DTW* to ensure efficiency. To address the demultiplexing problem, we designed a module based on the voting mechanism to parse the demultiplexing results from the clustering results. The usage of our method is presented in Additional file 1: S5. In the following, we give out the details of each step in the hybrid clustering and demultiplexing, where the detailed pseudocode for each step is given in Additional file 1: S3.

### Extract barcode information from raw data

Raw data comprises both the native nanopore signals and their corresponding DNA sequences. To successfully demultiplex the raw data, it is crucial to extract the barcode information accurately. To accomplish this, we have devised a heuristic scheme based on the distinctive characteristics of DNA libraries (refer to Fig. 5A).

In our approach, we begin by analyzing the native nanopore signal. We employ the s*emi-global dynamic time warping* algorithm to identify the position of the adapter signal within the signal. The tail position of the adapter signal serves as the starting point for the barcode signal. By leveraging this information, we are able to locate the barcode signal within the nanopore signal accurately. The determination of the barcode signal’s position takes into account the length of the barcode sequence and the sampling rate of the nanopore signal.

Similarly, for the DNA sequence, we utilize the *Edlib* to identify the position of the adapter sequence within the sequence. Subsequently, we determine the position of the barcode sequence based on its length. Assuming that the standard adapter sequence has a length of *n*, if the edit distance between the standard adapter sequence and the DNA sequence exceeds 0.45 times *n* (using local alignment), the barcode label of the sequence is deemed ambiguous and marked as “unclassified”.

All the sequences (signals) that we extract, both from the nanopore signals and DNA sequences, are referred to as pseudo-barcoded sequences (signals). These pseudo-barcoded sequences (signals) are utilized for subsequent clustering and demultiplexing stages.

### Initial clustering

**Fig. 7:**
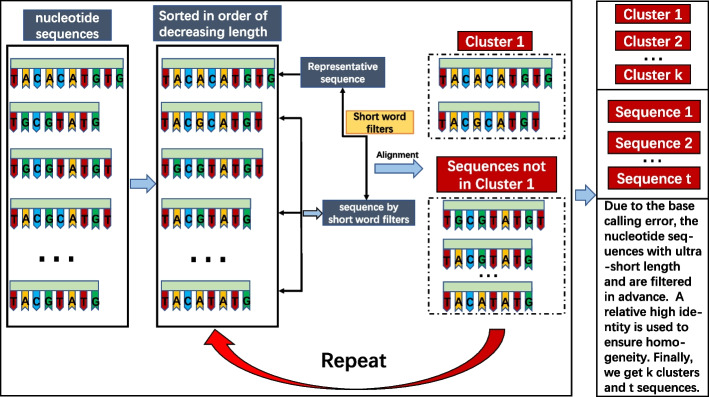
The workflow of initial clustering

We utilize a nucleobase-based greedy algorithm to generate homogeneous initial clustering, in which the process is similar to the ones in CD-HIT. Figure 7 describes the detailed workflow. Firstly, the sequences are sorted in descending order of the sequence length. The longest sequence is assumed to be the representative sequence of a cluster. A short word filter [13] is applied to reduce the comparison in pairwise alignment. Here, each selected sequence is compared with the existing representative sequences. If the similarity between the selected sequence and a representative sequence is higher than the threshold, the selected sequence will be merged into the cluster of the representative sequence. Otherwise, the selected sequence becomes a new representative sequence. Repeating this process until all the sequences are visited, resulting in a number of clusters and a set of ultra-short sequences that do not belong to any cluster. Finally, A verification mechanism is used to check the homogeneity of the clusters and retrieve the ultra-short sequences which are misclassified.

With a high enough threshold (e.g., identity $\geqslant$ 95%), the initial clustering is able to quickly generate clusters with high homogeneity, where these initial clusters can be considered as completely correct, to significantly reduce the pair comparison in further signal-similarity based clustering.

### Threshold determination

After obtaining the initial clustering result, we need to further refine it according to the raw signal information. The refinement of the initial clusters depends on the merging threshold, which is critical to the final demultiplexing accuracy. Because of the initial clusters’ high homogeneity, the merge threshold is possible to be determined from the initial clustering result.

Once the initial clustering is complete, we obtain the representative units of good clusters and their corresponding nanopore signals. All clusters are sorted in descending order based on their size, and the clusters ranked top-($0.01 \times |clusters|$) are called good clusters. |*clusters*| refers to the number of clusters, the size of a cluster with rank exactly $0.01 \times |clusters|$ is defined as *GoodIndex*. We calculate pairwise DTW distances of all nanopore signals and set the threshold as the average of the maximum and minimum distances divided by a constant value k (default is 4).

### Cluster merging and refinement

#### Cluster merging

For a certain multiplex sequencing configuration, it is possible to estimate the minimal set size of a cluster. Here, we define an initial cluster with set size larger than *GoodIndex* as a good cluster and denote $GoodClusterSet =\left\{ C_{1},C_{2},...,C_{M}\right\}$ as the set of good clusters of the initial clustering result, where *M* is the number of good clusters and $\{C_{i}\}$ is sorted in the descending order according to their cardinality.

For each $C_{i}$, we randomly select *K* raw signal sequences within $C_{i}$, and record these signals as $\{sig_{i_{k}}\}_{k=1,2,...,K}$, where *K* must satisfy

$$\begin{aligned} K <\min \{ \left| C_{i}\right| \}_ {i=1,2,...,M}. \end{aligned}$$

Every time we choose the top unvisited cluster $C_{i}$, i.e., the largest unvisited cluster in *GoodClusterSet*, as a query to compare with the other clusters ($i=1$ in the first time). We compare $\{sig_{i_{k}}\}$ with the sampled *K* raw signals $\{sig_{m_{k}}\}$ from the rest clusters ($1<m<M$) by the DTW distance. If for $\forall p,q,\ DTW(sig_{i_{p}},sig_{m_{q}}) <threshold$, $C_{m}$ is merged into $C_{i}$, and $GoodClusterSet = GoodClusterSet \backslash C_{m}$. Every time, the selected cluster $C_{i}$ is compared with the remaining clusters $\{C_{m}\}$ and is merged with all the clusters $C_{m}$ that satisfy the DTW distance constraint. We iteratively select the top unvisited cluster and make the cluster merging until all the clusters’ relationship has been checked.

#### Refinement 1

It should be noted that the *GoodClusterSet* has been changed during cluster merging. After the final merging, a set of refined clusters can be obtained, i.e., $GoodClusterSet' =\left\{ C_{1}',C_{2}',...,C_{T}'\right\} , T \le M$. The corresponding representative sequence set can be denoted as

$$\begin{aligned} ConSeqSet =\left\{ cseq_{1},cseq_{2},...,cseq_{T} \right\} . \end{aligned}$$

Given the representative sequence, its corresponding nanopore signal can also be obtained. Thus, with the representative sequences, a set of representative signal can be generated, which is denoted as

$$\begin{aligned} ConSigSet =\left\{ csig_{1},csig_{2},...,csig_{T}\right\} . \end{aligned}$$

The representative signal is utilized as standard reference to optimize the initial clustering results. For the sequences that are not in $C_{i},i = 1,2,...,M$, we get the corresponding raw signals of these sequences and calculate the DTW distance between these sequences and the representative signals in *ConSigSet*. For a given sequence, if the distance between this sequence’s raw signal and a representative signal is less than the *threshold*, the sequence is merged to the representative signal’s corresponding cluster.

#### Refinement 2

After the above steps, there are still some sequences that have not been classified. We get the raw signals of these sequences and make the following process: first, randomly select a sequence to generate a new cluster $C_{new}$, where the selected sequence is the representative sequence of $C_{new}$, denoted by $seq_{new}$. Calculate the DTW distance between the raw signal of $seq_{new}$ and the raw signal of the remaining sequences. If the distance is less than *threshold*, add the corresponding sequence to $C_{new}$. Repeat the process until all the sequences are visited.

Figure 1 illustrates an example for the merging and refinement process of 30 sequences from two cells. The clustering accuracy is continuously improved with the utilization of all the information residing in the nucleobase sequences and raw signals.

### Demultiplexing module based on voting mechanism

We performed demultiplexing on each cluster obtained from the hybrid clustering algorithm (as shown in Fig. 1D). To achieve this, we followed a specific procedure.

Given a cluster set, we initially selected the first k elements (with a default value of 5) corresponding to *k* pseudo-barcoded signals. For these selected signals, we computed the DTW distance matrix between them and all the standard barcode signals.

Next, we determined the row index of the minimum value in each column of the DTW distance matrix, resulting in a k-dimensional vector. This vector captures the closest match for each pseudo-barcoded signal among the standard barcode signals.

Finally, we calculated the mode of the k-dimensional vector, which represents the most frequent value in the vector. This mode value serves as the final demultiplexing result for the cluster. In other words, it represents the demultiplexing outcome for each sequence within the cluster.

Specifically, we identify sequences with ambiguous barcode labels using a straightforward and predetermined DTW distance threshold. The first cluster obtained from the clustering results is often of high quality, serving as a basis for generating a DTW distance threshold. Here is the refined description of the process. Firstly, 100 sequences are randomly selected from the cluster. Secondly, the DTW distance matrix is calculated for their corresponding pseudo-barcode signals. Next, the average value of the matrix elements is computed and referred to as “mean.” Finally, a threshold is set to *c* times the mean, with a default value of *c* as 1.65. In the case of a cluster set containing only one sequence, the following criterion is applied: If the minimum DTW distance between the standard barcode signal and the pseudo-barcode signal corresponding to this sequence exceeds the threshold, the barcode label of the sequence is considered ambiguous and marked as “unclassified.”

### GPU-accelerated DTW

**Fig. 8:**
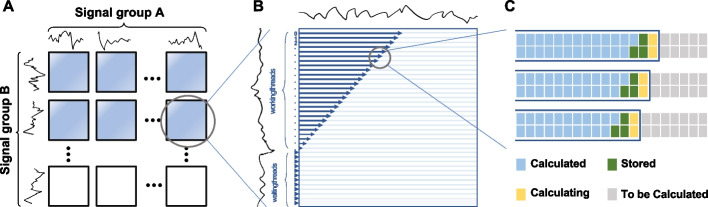
GPU-supported parallelization strategy. **A** Different signal pairs’ DTW distances are computed simultaneously within a CUDA block. **B** Each DTW matrix is calculated with multi-threads, where a register variable is used to control the wait relationships of all threads. **C** A thread can simultaneously compute multiple rows, where the values needed for the yellow cell (to be computed) are already stored in the shared memory (green cell)

The most computational expensive part of HycDemux is the calculation of the tens of millions to hundreds of millions of DTW distances. Generally, the computational complexity of a DTW algorithm should be *O*(*mn*) if the algorithm is sequentially implemented, where *m* and *n* are the lengths of the compared sequences. However, with the development of graphics processing unit (GPU) for general purpose processing, CUDA (or Compute Unified Device Architecture) has been widely used to accelerate computational biology tasks [48–50]. Here, we propose a CUDA-based GPU-accelerated DTW to solve the speed problem, by combining a coarse-grained block-wise acceleration strategy and a fine-grained multi-thread acceleration strategy. Figure 8 describes the outline of our acceleration strategy.

#### Dependency analysis

**Fig. 9:**
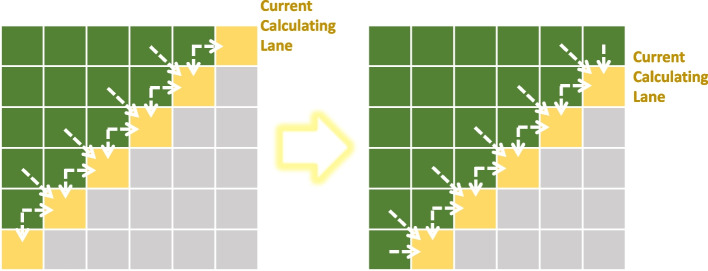
Data dependence within the calculation of a DTW matrix. The white dash arrow shows the dependence of element values if a certain element is to be calculated. Nevertheless, all the yellow elements have no dependence with each other, which enables their parallel calculation

Obviously, the DTW distance between different signals is totally independent. Thus, a coarse-grained block-wise parallel strategy is devised to calculate these DTW distances simultaneously within each CUDA block, as shown in Fig. 8A. The computation of DTW weight matrix could also be accelerated by CUDA. However, data dependence exists in the calculation of a DTW matrix, i.e., the calculation of position (*i*, *j*) in the DTW matrix needs the values in position $(i -1, j)$, $(i -1, j-1)$, and $(i, j-1)$. Here, considering the elements on a slash lane of a DTW matrix, these elements are independent with each other (Fig. 9). Thus, we change the calculation of DTW matrix from sequence order into slash-lane order and propose a fine-grained multi-thread parallel strategy to ensure the speed and accuracy, as shown in Fig. 8B.

#### Block-wise acceleration

Within a general GPU card with NVIDIA Turing architecture, up to a few million blocks are allowed to execute asynchronously and concurrently. As shown in Fig. 8A, each CUDA block is responsible for calculating one DTW distance. That is, millions of blocks could be initialized to calculate these DTW distances simultaneously, which makes the calculation extremely fast. In contrast, a multi-CPU server may only contain a few dozen cores, allowing the simultaneous calculation of only a few dozen DTW distances.

#### Multi-thread acceleration

Each DTW matrix is calculated by multiple threads lane by lane. Synchronize strategy is applied to ensure that the values needed by the current position have been calculated correctly. To control which columns should be calculated at a given time, we use a register variable *T* to serve as a timer. $\forall i\in \lbrace 0,1,2,...,n-1\rbrace$, the *i*th thread calculates the *i*th row (counting from 0), then the thread with thread number *t* needs to process the $(T-t)$th element of the row at time *T*. And the threads with thread number $c=T-t<0$ should wait in place until $T-c>0$. Figure 8B shows an example with $T=25$.

#### On-chip storage

Since a CUDA block contains 1024 threads at most but the longest signal length is up to $\sim$1500 (a barcode’s length is up to 145, while the corresponding signal is $8\sim 10$ times of the barcode sequence), we extended the algorithm to let a thread computes two DTW matrix rows at a time, which makes a block able to process 2048-length signal. Considering barcode sequences are not too long (such as 40 nt), the GPU card of the current Turing architecture can fully store data and perform calculations in on-chip memory (shared memory), which avoids the copy cost from the global memory and further accelerates the calculation. As shown in Fig. 8C, green cells represent the elements stored in shared memory, and the yellow cells are the elements being calculating. Actually, maximum amount of shared memory per block is 163 KB on NVIDIA Turing architecture, which provides the ability that one thread processes 9 DTW rows whose elements stored in single-precision float format.

### Supplementary information

**Additional file 1. S1.** Evaluation criteria. **S2.** Comparison experiment table of signal-similarity based clustering method and base space-based clustering method. **S3.** Comparison tables of hybrid clustering algorithm and three clustering tools. **S4.** Pseudo code about hybrid clustering algorithm. **S5.** Usage of our method. **Table S1.** A performance comparison of various clustering methods was conducted on a simulated dataset containing 50 clusters, with 2000 sequences of approximately 145bp in length. **Table S2.** A performance comparison of various clustering methods was conducted on a simulated dataset containing 100 clusters, with 2000 sequences of approximately 145bp in length. **Table S3.** A performance comparison of various clustering methods was conducted on a simulated dataset containing 20 clusters, with 2000 sequences of approximately 145bp in length. **Table S4.** A performance comparison of various clustering methods was conducted on a simulated dataset containing 100 clusters, with 2000 sequences of approximately 95bp in length. **Table S5.** A performance comparison of various clustering methods was conducted on a simulated dataset containing 50 clusters, with 2000 sequences of approximately 95bp in length. **Table S6.** A performance comparison of various clustering methods was conducted on a simulated dataset containing 20 clusters, with 2000 sequences of approximately 95bp in length. **Table S7.** A performance comparison of various clustering methods was conducted on a simulated dataset containing 100 clusters, with 2000 sequences of approximately 45bp in length. **Table S8.** A performance comparison of various clustering methods was conducted on a simulated dataset containing 50 clusters, with 2000 sequences of approximately 45bp in length. **Table S9.** A performance comparison of various clustering methods was conducted on a simulated dataset containing 20 clusters, with 2000 sequences of approximately 45bp in length. **Table S10.** Comparison of the performances of the three tools and our method on the simulation data set 1. **Table S11.** Comparison of the performances of the three tools and our method on the simulation data set 2. **Table S12.** Comparison of the performances of the three tools and our method on the simulation data set 3. **Table S13.** Comparison of the performances of the three tools and our method on the simulation data set 4. **Table S14.** Comparison of the performances of the three tools and our method on the simulation data set 5. **Table S15.** Comparison of the performances of the three tools and our method on the simulation data set 6.

**Additional file 2.** Review history.

## Data Availability

To evaluate our hybrid clustering algorithm, we utilized both simulated and real datasets. The simulated data sets (dataset S1 to S6) are accessible at the following URL: https://doi.org/10.5281/zenodo.8256481 [51]. The real dataset [52] are accessible at https://doi.org/10.5281/zenodo.8256500, obtained (details are in the Results) from the European Nucleotide Archive (ENA) under accession ERR2767931 [33] and *figshare*, can be accessed at https://ftp.sra.ebi.ac.uk/vol1/run/ERR276/ERR2767931/deepbinner_amplicon_fast5s.tar.gz and https://figshare.com/projects/Deepbinner/34223. Both the simulated and real datasets contain the extracted barcode signals and sequences, which are essential for the direct evaluation of our hybrid clustering algorithm in HycDemux. All reads with barcodes (considered as positive samples) in datasets D1 to D7 can be accessed via the following URL: https://doi.org/10.5281/zenodo.8264231 [53]. Additionally, the reads [54] without true barcodes (considered as negative samples) in datasets D1 to D7 are accessible at https://doi.org/10.5281/zenodo.8260510. Moreover, the reads [55] in datasets DB4 to DB7 are accessible at https://doi.org/10.5281/zenodo.8260583. The nanopore signals in datasets D1 to D7 (DB4 to DB7) have been divided into multiple parts for convenience. Specifically, dataset D1 can be accessed at https://doi.org/10.5281/zenodo.8264226 [56], while dataset D2 can be accessed at https://doi.org/10.5281/zenodo.8264249 [57]. D3 can be accessed at four separate URLs: https://doi.org/10.5281/zenodo.8256994 [58], https://doi.org/10.5281/zenodo.8264210 [59], https://doi.org/10.5281/zenodo.8260102 [60], and https://doi.org/10.5281/zenodo.8264137 [61]. The datasets D4 to D7 (DB4 to DB7) are available at the following URLs: https://doi.org/10.5281/zenodo.8266227 [62], https://doi.org/10.5281/zenodo.8266246 [63], https://doi.org/10.5281/zenodo.8266248 [64], https://doi.org/10.5281/zenodo.8266251 [65], https://doi.org/10.5281/zenodo.8266225 [66], https://doi.org/10.5281/zenodo.8266223 [67], https://doi.org/10.5281/zenodo.8266221 [68], https://doi.org/10.5281/zenodo.8264285 [69], https://doi.org/10.5281/zenodo.8266219 [70], and https://doi.org/10.5281/zenodo.8266213 [71]. Negative sample signals of D1 to D7 (DB4 to DB7) can be accessed at https://doi.org/10.5281/zenodo.8260534 [72]. The HycDemux software is available on GitHub at https://github.com/junhaiqi/Hybrid_clustering.git [73] under the GNU General Public License v3.0. Additionally, the source code for HycDemux has been deposited at https://doi.org/10.5281/zenodo.8260659 [74].
