# Supplementary material for: HycDemux: a hybrid unsupervised approach for accurate barcoded sample demultiplexing in nanopore sequencing
Source: Genome Biol. 2023 Oct 5;24:222. doi: 10.1186/s13059-023-03053-1 (PMC10552309; doi:10.1186/s13059-023-03053-1)
Supplement: Supplementary file 1 — Additional file 1. S1. Evaluation criteria. S2. Comparison experiment table of signal-similarity based clustering method and base space-based clustering method. S3. Comparison tables of hybrid clustering algorithm and three clustering tools. S4. Pseudo code about hybrid clustering algorithm. S5. Usage of our method. Table S1. A performance comparison of various clustering methods was conducted on a simulated dataset containing 50 clusters, with 2000 sequences of approximately 145bp in length. Table S2. A performance comparison of various clustering methods was conducted on a simulated dataset containing 100 clusters, with 2000 sequences of approximately 145bp in length. Table S3. A performance comparison of various clustering methods was conducted on a simulated dataset containing 20 clusters, with 2000 sequences of approximately 145bp in length. Table S4. A performance comparison of various clustering methods was conducted on a simulated dataset containing 100 clusters, with 2000 sequences of approximately 95bp in length. Table S5. A performance comparison of various clustering methods was conducted on a simulated dataset containing 50 clusters, with 2000 sequences of approximately 95bp in length. Table S6. A performance comparison of various clustering methods was conducted on a simulated dataset containing 20 clusters, with 2000 sequences of approximately 95bp in length. Table S7. A performance comparison of various clustering methods was conducted on a simulated dataset containing 100 clusters, with 2000 sequences of approximately 45bp in length. Table S8. A performance comparison of various clustering methods was conducted on a simulated dataset containing 50 clusters, with 2000 sequences of approximately 45bp in length. Table S9. A performance comparison of various clustering methods was conducted on a simulated dataset containing 20 clusters, with 2000 sequences of approximately 45bp in length. Table S10. Comparison of the performances of the three tools and [file 13059_2023_3053_MOESM1_ESM.pdf]

Additional file 1: Supplementary material for "HycDemux: A hybrid unsupervised approach for accurate barcoded sample demultiplexing in nanopore sequencing"

Renmin Han[1,†], Junhai Qi[1,2,†], Yang Xue[1], Xiujuan Sun[4], Fa Zhang[4,*], Xin Gao[5,*], Guojun Li[1,*]

S1 Evaluation criteria

We applied the following six evaluation indexes in evaluating hybrid clustering algorithm and related tools: (1) adjusted mutual information(AMI), (2) Fowlkes-Mallows scores(FMI), (3) accuracy(ACC), (4) Homogeneity(HOMO), (5)completeness(COMP), (6)time. We know the cluster distribution of each dataset, so we use these indexes to measure the effectiveness of clustering. The implications of indexes are described in the following paragraphs.

Given sequence set $X = \{x_1, x_2, ..., x_N\}$. For $x_i \in X$, the corresponding label for x_i is u_i, this means that x_i belongs to the u_i-th cluster, $i \in \{1, 2, ..., N\}$. We can get the true label set $P = \{u_1, u_2, ..., u_N\}$. If X has R clusters, then

$$\max_{1<i<N} P = R$$

we define $U = \{U_1, U_2, ..., U_R\}$. U_t represents the set of data numbers belonging to t-th cluster, $t \in \{1, 2, ..., R\}$. For instance, $U_1 = \{1, 2, 5\}$, this means that x_1, x_2, x_5 belongs to the first cluster.

By using the clustering algorithm, the actual label set can be obtained. we define $V = \{V_1, V_2, ..., V_C\}$. This means that there are C clusters in the actual clustering results, where V_j represents the data number set belonging to the j-th cluster, $j \in \{1, 2, ..., C\}$.

We define metric $M \in \mathbb{R}^{R\times C}$ is a contingency table of U and V. m_{ij} is a element of the i-th row, j-th column of M, $m_{ij} = \left|U_i \cap V_j\right|$. We define

$$MI(U, V) = \sum_{i=1}^{R} \sum_{j=1}^{C} p_{i,j} \cdot log\left(\frac{p_{i,j}}{p_i \times p_j}\right) \tag{1}$$

$$H(U) = -\sum_{i=1}^{R} p_i \cdot log\ p_i \tag{2}$$

$$p_i = \frac{|U_i|}{N},\ p_j = \frac{\left|U_j\right|}{N} \tag{3}$$

$$p_{i,j} = \frac{m_{ij}}{N} \tag{4}$$

$$\mathbb{E}\{MI(U, V)\} = \sum_{i=1}^{R} \sum_{j=1}^{C} \sum_{k=c}^{\min(a_i, b_i)} A \tag{5}$$

$$c = \max(1, a_i + b_i - N) \tag{6}$$

$$A = \frac{k}{N} \cdot log\left(\frac{N \times k}{a_i \times b_i}\right) \frac{a_i! b_j! (N - a_i)! (N - b_j)!}{N! k! (a_i - k)! (b_j - k)! (N - a_i - b_j + k)!} \tag{7}$$

$$a_i = \sum_{j=1}^{C} m_{ij},\ b_j = \sum_{i=1}^{R} m_{ij} \tag{8}$$

$$AMI(U, V) = \frac{MI(U, V) - \mathbb{E}\{MI(U, V)\}}{\frac{(H(U)+H(V))}{2} - \mathbb{E}\{MI(U, V)\}} \tag{9}$$

(1)-(9) show the calculation process of AMI. we define M, T, P, Q is the set of point pairs (x_i, x_j). The point pair in M satisfies the condition: $\exists k, t,\ s.t.\ (x_i, y_j) \in V_k$, and $(x_i, y_j) \in U_t$. The point pair in T satisfies the condition: $\exists k,\ s.t.\ (x_i, y_j) \in V_k$, but for $\forall t,\ (x_i, y_j) \notin U_t$. The point pair in P satisfies the condition: for $\forall k,\ (x_i, y_j) \notin V_k$, but $\exists t,\ s.t.\ (x_i, y_j) \in U_t$. The point pair in Q satisfies the condition: for $\forall k,\ (x_i, y_j) \notin V_k$, and for $\forall t,\ (x_i, y_j) \notin U_t$. $k \in \{1, 2, ..., R\}$, $t \in \{1, 2, ..., C\}$. (10)-(11) show the calculation process of FMI and ACC.

$$FMI = \sqrt{\frac{M}{M + T} \cdot \frac{M}{M + P}} \tag{10}$$

$$ACC = \frac{M+Q}{N} \tag{11}$$

AMI, FMI and ACC reflect the similarity between the clustering label set generated by clustering results and the real clustering label set, so they can represent the clustering effect. HOMO is an index that reflects the local correctness of clustering results. For example, $U_1 = \{1, 2, 5, 6\}$, $V_2 = \{1, 5, 6\}$, the V_2 of homogeneity is 100%. $COMP$ reflects the completeness of the cluster obtained by clustering results. For V_2, although its $HOMO$ is 100%, completeness is not, because x_6 not in V_2. (12)-(16) show the calculation process of $HOMO$ and $COMP$.

$$H(U) = -\sum_{i=1}^{R} \frac{|U_i|}{N} \cdot log\left(\frac{|U_i|}{N}\right) \tag{12}$$

$$H(V) = -\sum_{i=1}^{C} \frac{|V_i|}{N} \cdot log\left(\frac{|V_i|}{N}\right) \tag{13}$$

$$H(U|V) = -\sum_{i=1}^{R}\sum_{j=1}^{C} \frac{m_{ij}}{N} \cdot log\left(\frac{m_{ij}}{N}\right) \tag{14}$$

$$H(V|U) = -\sum_{i=1}^{C}\sum_{j=1}^{R} \frac{m_{ij}}{N} \cdot log\left(\frac{m_{ij}}{N}\right) \tag{15}$$

$$HOMO = 1 - \frac{H(U|V)}{H(u)},\ COMP = 1 - \frac{H(V|U)}{H(V)} \tag{16}$$

S2 Comparison experiment table of signal-similarity based clustering method and base space-based clustering method

In order to show that the signal-similarity based clustering method is better than the base space-based clustering method, we have generated a total of 9 data sets. The results of one of the data sets have been shown in the paper. The following eight tables show the results of the remaining 8 data sets.

Data type	Tools, methods and evaluation index							
	Tool, Method	Identity(%)	AMI(%)	FMI(%)	ACC(%)	HOMO(%)	COMP(%)	Time(min:sec)
Base	DNACLUST	80	82.71	74.6	67.6	100.0	79.47	0:01.28
	MeShClust	80	91.8	84.05	85.4	95.51	91.67	0:24.53
	CD-HIT	80	92.39	87.15	82.75	99.69	89.43	0:00.20
	UCLUST	80	79.61	71.52	64.0	100.0	77.01	0:00.10
	DNACLUST	85	64.04	53.3	43.15	100.0	67.56	0:01.85
	MeShClust	85	92.36	90.23	87.35	99.8	89.39	0:34.42
	CD-HIT	85	83.46	75.44	69.25	100.0	80.11	0:00.18
	UCLUST	85	60.68	51.74	41.2	100.0	66.07	0:00.34
	DNACLUST	90	33.8	28.67	20.8	100.0	57.38	0:02.12
	MeShClust	90	74.91	70.66	64.45	99.49	73.98	0:07.36
	CD-HIT	90	60.98	51.29	40.85	100.0	66.18	0:00.29
	UCLUST	90	30.77	27.08	19.1	100.0	56.69	0:00.20
Signal	K-means	-	100.00	100.00	100.00	100.00	100.00	1733:1.19
	Hierarchical clustering	-	100.00	100.00	100.00	100.00	100.00	1733:4.65
	Spectral clustering	-	100.00	100.00	100.00	100.00	100.00	1733:3.9

Table S1: Performance comparison of different clustering methods on a simulated dataset. The dataset is simulated by DeepSimulator, using 50 categories of 145bp nucleotide sequences as templates with 40 random seeds. Totally, 2000 nanopore raw current signals are simulated, with 50 clusters on the dataset and 40 sequences for each cluster.

Data type	Tools, methods and evaluation index							
	Tool, Method	Identity(%)	AMI(%)	FMI(%)	ACC(%)	HOMO(%)	COMP(%)	Time(min:sec)
Base	DNACLUST	80	83.89	77.81	72.30	100	85.03	00:02.11
	MeShClust	80	92.55	87.81	87.95	98.01	93.35	00:14.26
	CD-HIT	80	92.18	87.46	84.25	99.8	91.75	00:00.13
	UCLUST	80	78.91	71.04	63.80	100	81.84	00:00.10
	DNACLUST	85	65.22	57.88	49.15	100	75.12	00:02.58
	MeShClust	85	90.54	87.80	85.45	99.65	90.39	00:22.16
	CD-HIT	85	82.19	75.31	68.3	100	83.88	00:00.15
	UCLUST	85	59.02	51.49	41.95	100	72.78	00:00.23
	DNACLUST	90	31.76	29.42	22.25	100	65.54	00:02.61
	MeShClust	90	68.96	65.88	58.25	100	76.76	00:56.66
	CD-HIT	90	58.59	50.94	42.90	100	72.63	00:00.20
	UCLUST	90	28.62	27.59	21.00	100	64.93	00:00.19
Signal	K-means	-	100.00	100.00	100.00	100.00	100.00	1732:54.24
	Hierarchical clustering	-	100.00	100.00	100.00	100.00	100.00	1732:57.50
	Spectral clustering	-	100.00	100.00	100.00	100.00	100.00	1732:55.75

Table S2: Performance comparison of different clustering methods on a simulated dataset. The dataset is simulated by DeepSimulator, using 100 categories of 145bp nucleotide sequences as templates with 20 random seeds. Totally, 2000 nanopore raw current signals are simulated, with 100 clusters on the dataset and 20 sequences for each cluster.

Data type	Tools, methods and evaluation index							
Base	Tool, Method	Identity(%)	AMI(%)	FMI(%)	ACC(%)	HOMO(%)	COMP(%)	Time(min:sec)
	DNACLUST	80	83.86	77.16	72.8	97.69	77.18	0:00.71
	MeShClust	80	88.28	80.61	80.9	91.34	87.13	0:16.76
	CD-HIT	80	90.85	82.81	78.85	97.69	86.22	0:00.19
	UCLUST	80	75.83	63.15	56.0	97.69	68.13	0:00.20
	DNACLUST	85	66.57	54.55	46.5	97.69	61.02	0:01.07
	MeShClust	85	89.67	83.73	81.5	94.97	86.78	0:01.06
	CD-HIT	85	83.51	76.03	72.2	97.69	76.66	0:00.17
	UCLUST	85	58.85	46.51	36.4	97.69	55.63	0:00.19
	DNACLUST	90	37.24	28.84	21.65	97.69	46.73	0:01.40
	MeShClust	90	81.99	79.69	74.9	97.69	75.58	0:49.25
	CD-HIT	90	65.33	54.56	47.65	97.69	60.27	0:00.24
	UCLUST	90	31.55	25.65	15.75	97.69	45.01	0:00.18
Signal	K-means	-	100.00	100.00	100.00	100.00	100.00	1733:1.0
	Hierarchical clustering	-	100.00	100.00	100.00	100.00	100.00	1733:4.83
	Spectral clustering	-	100.00	100.00	100.00	100.00	100.00	1733:2.30

Table S3: Performance comparison of different clustering methods on a simulated dataset. The dataset is simulated by DeepSimulator, using 20 categories of 145bp nucleotide sequences as templates with 100 random seeds. Totally, 2000 nanopore raw current signals are simulated, with 20 clusters on the dataset and 100 sequences for each cluster.

Data type	Tools, methods and evaluation index							
Base	Tool, Method	Identity(%)	AMI(%)	FMI(%)	ACC(%)	HOMO(%)	COMP(%)	Time(min:sec)
	DNACLUST	80	70.47	63.8	55.15	100.0	77.42	0:01.62
	MeShClust	80	83.16	77.06	75.2	97.92	85.42	0:14.51
	CD-HIT	80	84.19	77.27	70.65	100.0	85.23	0:00.13
	UCLUST	80	65.69	58.07	49.2	100.0	75.31	0:00.08
	DNACLUST	85	51.62	46.75	37.85	100.0	70.4	0:01.72
	MeShClust	85	79.36	75.96	69.65	99.71	82.24	0:01.17
	CD-HIT	85	69.48	61.63	53.0	100.0	76.95	0:00.15
	UCLUST	85	47.56	42.3	33.7	100.0	69.24	0:00.20
	DNACLUST	90	24.5	24.73	18.15	100.0	64.17	0:01.39
	MeShClust	90	55.69	52.44	43.2	100.0	71.69	0:44.08
	CD-HIT	90	46.62	40.99	32.55	100.0	68.98	0:00.12
	UCLUST	90	23.96	24.42	17.95	100.0	64.08	0:00.13
Signal	K-means	-	100.00	100.00	100.00	100.00	100.00	1099:59.21
	Hierarchical clustering	-	100.00	100.00	100.00	100.00	100.00	1100:2.60
	Spectral clustering	-	100.00	100.00	100.00	100.00	100.00	1100:1.85

Table S4: Performance comparison of different clustering methods on a simulated dataset. The dataset is simulated by DeepSimulator, using 100 categories of 95bp nucleotide sequences as templates with 20 random seeds. Totally, 2000 nanopore raw current signals are simulated, with 100 clusters on the dataset and 20 sequences for each cluster.

Data type	Tools, methods and evaluation index							
	Tool, Method	Identity(%)	AMI(%)	FMI(%)	ACC(%)	HOMO(%)	COMP(%)	Time(min:sec)
Base	DNACLUST	80	69.82	60.87	50.6	100.0	70.61	0:01.18
	MeShClust	80	86.58	80.52	80.25	96.89	84.56	0:42.02
	CD-HIT	80	84.33	77.3	69.6	99.94	80.93	0:00.14
	UCLUST	80	69.4	61.58	50.6	99.94	70.42	0:00.08
	DNACLUST	85	52.11	42.53	32.8	100.0	62.66	0:01.36
	MeShClust	85	82.96	79.17	75.05	98.77	80.35	0:56.57
	CD-HIT	85	71.29	62.03	51.9	100.0	71.44	0:00.17
	UCLUST	85	51.63	43.14	32.65	100.0	62.51	0:00.21
	DNACLUST	90	28.25	25.21	17.45	100.0	56.15	0:01.17
	MeShClust	90	62.8	59.76	51.5	100.0	67.21	0:14.53
	CD-HIT	90	51.58	43.58	33.55	100.0	62.51	0:00.17
	UCLUST	90	28.49	25.48	18.05	100.0	56.2	0:00.13
Signal	K-means	-	100.00	100.00	100.00	100.00	100.00	1100:2.17
	Hierarchical clustering	-	100.00	100.00	100.00	100.00	100.00	1100:5.57
	Spectral clustering	-	100.00	100.00	100.00	100.00	100.00	1100:4.9

Table S5: Performance comparison of different clustering methods on a simulated dataset. The dataset is simulated by DeepSimulator, using 50 categories of 95bp nucleotide sequences as templates with 100 random seeds. Totally, 2000 nanopore raw current signals are simulated, with 50 clusters on the dataset and 40 sequences for each cluster.

Data type	Tools, methods and evaluation index							
	Tool, Method	Identity(%)	AMI(%)	FMI(%)	ACC(%)	HOMO(%)	COMP(%)	Time(min:sec)
Base	DNACLUST	80	63.33	50.95	41.25	100.0	58.1	0:00.99
	MeShClust	80	92.58	90.55	89.15	99.82	87.47	0:01.09
	CD-HIT	80	80.13	68.99	61.65	100.0	71.35	0:00.14
	UCLUST	80	63.5	55.64	45.75	100.0	58.61	0:00.09
	DNACLUST	85	46.16	37.39	28.0	100.0	50.02	0:01.30
	MeShClust	85	81.66	79.61	77.05	99.84	73.92	0:01.11
	CD-HIT	85	65.38	52.23	43.55	100.0	59.33	0:00.21
	UCLUST	85	45.94	37.3	27.7	100.0	49.99	0:00.24
	DNACLUST	90	24.32	19.72	13.3	100.0	43.61	0:01.11
	MeShClust	90	53.95	52.55	46.05	100.0	54.13	0:01.82
	CD-HIT	90	44.91	37.66	28.35	100.0	49.63	0:00.21
	UCLUST	90	24.94	21.02	13.8	100.0	43.78	0:00.15
Signal	K-means	-	100.00	100.00	100.00	100.00	100.00	1099:59.57
	Hierarchical clustering	-	100.00	100.00	100.00	100.00	100.00	1100:3.26
	Spectral clustering	-	100.00	100.00	100.00	100.00	100.00	1100:0.80

Table S6: Performance comparison of different clustering methods on a simulated dataset. The dataset is simulated by DeepSimulator, using 20 categories of 95bp nucleotide sequences as templates with 100 random seeds. Totally, 2000 nanopore raw current signals are simulated, with 20 clusters on the dataset and 100 sequences for each cluster.

Data type	Tools, methods and evaluation index							
	Tool, Method	Identity(%)	AMI(%)	FMI(%)	ACC(%)	HOMO(%)	COMP(%)	Time(min:sec)
Base	DNACLUST	80	35.81	31.42	24.15	99.94	66.37	0:01.10
	MeShClust	80	27.3	19.39	22.7	94.08	63.77	0:57.47
	CD-HIT	80	55.21	45.06	38.2	98.53	71.48	0:00.10
	UCLUST	80	33.04	30.31	23.15	100.0	65.8	0:00.04
	DNACLUST	85	21.53	22.14	17.4	99.95	63.65	0:00.90
	MeShClust	85	18.04	12.04	16.1	95.92	62.36	0:01.58
	CD-HIT	85	39.98	34.04	26.5	99.8	67.29	0:00.07
	UCLUST	85	22.9	23.54	18.0	100.0	63.89	0:00.21
	DNACLUST	90	10.48	13.82	11.6	100.0	61.96	0:00.64
	MeShClust	90	13.76	5.97	10.5	70.95	59.15	1:09.52
	CD-HIT	90	21.74	22.15	17.0	99.92	63.68	0:00.07
	UCLUST	90	11.94	15.76	12.3	100.0	62.17	0:00.05
Signal	K-means	-	100.00	100.00	100.00	100.00	100.00	267:4.5
	Hierarchical clustering	-	100.00	100.00	100.00	100.00	100.00	267:7.33
	Spectral clustering	-	100.00	100.00	100.00	100.00	100.00	267:7.18

Table S7: Performance comparison of different clustering methods on a simulated dataset. The dataset is simulated by DeepSimulator, using 100 categories of 45bp nucleotide sequences as templates with 20 random seeds. Totally, 2000 nanopore raw current signals are simulated, with 100 clusters on the dataset and 20 sequences for each cluster.

Data type	Tools, methods and evaluation index							
	Tool, Method	Identity(%)	AMI(%)	FMI(%)	ACC(%)	HOMO(%)	COMP(%)	Time(min:sec)
Base	DNACLUST	80	39.38	30.28	22.35	99.98	58.72	0:00.80
	MeShClust	80	36.22	21.06	24.55	84.23	55.57	0:56.89
	CD-HIT	80	59.5	44.05	35.6	98.66	65.4	0:00.09
	UCLUST	80	35.8	27.86	20.3	99.98	57.82	0:00.05
	DNACLUST	85	24.69	21.63	15.4	99.98	55.4	0:00.74
	MeShClust	85	27.94	13.41	18.05	85.46	53.81	0:56.91
	CD-HIT	85	43.61	33.06	25.2	99.74	59.86	0:00.09
	UCLUST	85	26.52	22.97	16.25	100.0	55.77	0:00.26
	DNACLUST	90	11.66	12.13	8.85	99.98	53.11	0:00.60
	MeShClust	90	17.41	10.37	12.45	94.56	53.07	1:18.35
	CD-HIT	90	25.87	22.46	16.2	99.83	55.61	0:00.09
	UCLUST	90	13.23	14.6	10.35	100.0	53.37	0:00.06
Signal	K-means	-	100.00	100.00	100.00	100.00	100.00	266:56.94
	Hierarchical clustering	-	100.00	100.00	100.00	100.00	100.00	267:0.47
	Spectral clustering	-	100.00	100.00	100.00	100.00	100.00	266:59.2

Table S8: Performance comparison of different clustering methods on a simulated dataset. The dataset is simulated by DeepSimulator, using 50 categories of 45bp nucleotide sequences as templates with 40 random seeds. Totally, 2000 nanopore raw current signals are simulated, with 50 clusters on the dataset and 40 sequences for each cluster.

Data type	Tools, methods and evaluation index							
Base	Tool, Method	Identity(%)	AMI(%)	FMI(%)	ACC(%)	HOMO(%)	COMP(%)	Time(min:sec)
	DNACLUST	80	38.55	25.87	17.85	99.95	47.09	0:00.73
	MeShClust	80	49.63	33.88	31.8	87.87	50.16	0:01.15
	CD-HIT	80	58.78	42.73	31.7	99.46	55.15	0:00.10
	UCLUST	80	37.64	26.5	19.0	100.0	46.9	0:00.04
	DNACLUST	85	25.32	16.95	11.7	100.0	43.68	0:00.70
	MeShClust	85	39.23	26.4	24.7	90.22	46.44	0:57.44
	CD-HIT	85	44.24	30.39	21.0	100.0	48.97	0:00.10
	UCLUST	85	26.89	19.71	13.1	100.0	44.1	0:00.18
	DNACLUST	90	12.49	9.48	5.95	100.0	41.22	0:00.52
	MeShClust	90	21.03	11.31	13.45	84.77	40.79	0:57.63
	CD-HIT	90	28.04	20.64	13.7	99.98	44.37	0:00.10
	UCLUST	90	15.62	13.1	7.25	100.0	41.81	0:00.06
Signal	K-means	-	100.00	100.00	100.00	100.00	100.00	267:11.76
	Hierarchical clustering	-	100.00	100.00	100.00	100.00	100.00	267:15.47
	Spectral clustering	-	100.00	100.00	100.00	100.00	100.00	267:13.18

Table S9: Performance comparison of different clustering methods on a simulated dataset. The dataset is simulated by DeepSimulator, using 20 categories of 45bp nucleotide sequences as templates with 100 random seeds. Totally, 2000 nanopore raw current signals are simulated, with 20 clusters on the dataset and 100 sequences for each cluster.

S3 Comparison tables of hybrid clustering algorithm and three clustering tools

In order to compare the performance of the hybrid clustering algorithm with other clustering tools, we generated a total of 12 simulated data sets. In the paper, we show the performance of the hybrid clustering algorithm and the other three clustering tools on six data sets. The following 6 tables show their performance on the remaining 6 data sets.

Table S10: Comparison of the performances of the three tools and our method on the simulation data set 1

Tool	Identity(%)	AMI(%)	FMI(%)	ACC(%)	HOMO(%)	COMP(%)	Time(min:sec)
MeShClust	80.0	59.63	34.24	43.28	62.63	62.00	0:28.35
CD-HIT	80.0	77.54	65.52	55.38	100.00	65.83	0:00.23
UCLUST	80.0	63.52	50.81	40.70	100.00	53.54	0:00.13
MeShClust	85.0	61.06	39.73	49.54	73.00	56.67	0:25.51
CD-HIT	85.0	70.32	57.85	47.33	100.00	59.00	0:00.27
UCLUST	85.0	55.73	42.84	31.91	100.00	48.68	0:00.18
MeShClust	90.0	65.04	53.30	54.65	81.56	60.18	0:01.71
CD-HIT	90.0	57.21	42.73	33.09	100.00	49.60	0:00.40
UCLUST	90.0	44.28	30.54	20.84	100.00	43.04	0:00.28
MeShClust	95.0	56.43	42.29	36.28	96.43	49.60	0:02.02
CD-HIT	95.0	38.66	27.68	19.31	100.00	41.18	0:00.46
UCLUST	95.0	30.64	20.70	13.96	100.00	38.47	0:00.28
Hybrid clustering	—	98.46	98.13	97.83	100.00	97.00	0:3.60

[1] simulation data set 1 was obtained by DeepSimulator.

[2] Ten thousand sequences comprise this data set. Sequence lengths about 45 base pairs. The dataset has 20 classes. The performances were compared using these four identity scores: 80%, 85%, 90%, 95%.

Table S11: Comparison of the performances of the three tools and our method on the simulation data set 2

Tool	Identity(%)	AMI(%)	FMI(%)	ACC(%)	HOMO(%)	COMP(%)	Time(min:sec)
MeShClust	80.0	57.02	30.87	36.17	76.45	56.45	0:01.41
CD-HIT	80.0	78.77	63.25	53.18	100.00	70.91	0:00.23
UCLUST	80.0	61.72	44.52	34.04	100.00	58.98	0:00.18
MeShClust	85.0	44.47	15.27	27.62	62.47	52.13	0:01.95
CD-HIT	85.0	68.40	51.89	41.57	100.00	62.95	0:00.34
UCLUST	85.0	52.73	37.47	27.27	100.00	54.85	0:00.22
MeShClust	90.0	52.18	28.63	27.48	83.61	53.11	0:48.08
CD-HIT	90.0	52.94	37.71	28.07	100.00	54.96	0:00.41
UCLUST	90.0	37.94	25.82	17.06	100.00	49.78	0:00.28
MeShClust	95.0	47.73	30.23	24.28	94.09	52.25	0:02.43
CD-HIT	95.0	30.78	20.93	13.29	100.00	47.91	0:00.46
UCLUST	95.0	22.90	16.36	10.07	100.00	46.18	0:00.33
Hybrid clustering	—	97.17	96.51	95.32	100.00	94.59	0:04.84

[1] simulation data set 2 was obtained by DeepSimulator.

[2] Ten thousand sequences comprise this data set. Sequence lengths about 45 base pairs. The dataset has 50 classes. The performances were compared using these four identity scores: 80%, 85%, 90%, 95%.

Table S12: Comparison of the performances of the three tools and our method on the simulation data set 3

Tool	Identity(%)	AMI(%)	FMI(%)	ACC(%)	HOMO(%)	COMP(%)	Time(min:sec)
MeShClust	80.0	60.81	37.00	46.23	71.67	57.45	0:16.06
CD-HIT	80.0	82.74	73.33	67.20	100.00	72.06	0:00.30
UCLUST	80.0	66.67	51.05	42.10	100.00	55.61	0:00.29
MeShClust	85.0	64.76	54.49	53.82	88.07	57.03	0:56.34
CD-HIT	85.0	73.72	61.58	54.61	100.00	62.10	0:00.40
UCLUST	85.0	54.78	36.51	26.89	100.00	47.61	0:00.29
MeShClust	90.0	55.58	38.13	37.54	84.47	49.70	0:10.04
CD-HIT	90.0	59.26	45.16	35.78	100.00	50.70	0:00.75
UCLUST	90.0	40.58	24.62	16.91	100.00	41.41	0:00.40
MeShClust	95.0	53.03	47.33	38.55	95.97	48.00	0:02.07
CD-HIT	95.0	37.87	24.91	17.79	100.00	40.75	0:00.77
UCLUST	95.0	24.21	13.92	8.57	100.00	36.69	0:00.47
Hybrid clustering	—	99.07	99.17	99.11	100.00	98.18	0:05.40

[1] simulation data set 3 was obtained by DeepSimulator.

[2] Ten thousand sequences comprise this data set. Sequence lengths about 70 base pairs. The dataset has 20 classes. The performances were compared using these four identity scores: 80%, 85%, 90%, 95%.

Table S13: Comparison of the performances of the three tools and our method on the simulation data set 4

Tool	Identity(%)	AMI(%)	FMI(%)	ACC(%)	HOMO(%)	COMP(%)	Time(min:sec)
MeShClust	80.0	68.73	48.53	54.73	83.48	65.99	0:41.06
CD-HIT	80.0	86.98	76.74	70.70	100.00	79.77	0:00.29
UCLUST	80.0	72.56	58.82	47.41	100.00	66.00	0:00.23
MeShClust	85.0	65.49	41.89	46.36	83.96	62.67	0:01.45
CD-HIT	85.0	78.37	65.42	57.25	100.00	70.77	0:00.46
UCLUST	85.0	62.19	48.78	37.76	100.00	59.49	0:00.31
MeShClust	90.0	65.88	47.28	42.38	91.78	61.87	0:01.71
CD-HIT	90.0	64.45	50.87	41.38	100.00	60.79	0:00.66
UCLUST	90.0	47.05	35.28	24.26	100.00	52.81	0:00.45
MeShClust	95.0	63.06	45.94	38.72	94.85	59.91	0:56.56
CD-HIT	95.0	42.14	30.92	21.95	100.00	51.14	0:00.69
UCLUST	95.0	28.69	21.69	13.22	100.00	47.49	0:00.53
Hybrid clustering	—	94.34	92.27	91.39	100.00	90.19	0:05.84

[1] simulation data set 4 was obtained by DeepSimulator.

[2] Ten thousand sequences comprise this data set. Sequence lengths about 70 base pairs. The dataset has 50 classes. The performances were compared using these four identity scores: 80%, 85%, 90%, 95%.

Table S14: Comparison of the performances of the three tools and our method on the simulation data set 5

Tool	Identity(%)	AMI(%)	FMI(%)	ACC(%)	HOMO(%)	COMP(%)	Time(min:sec)
MeShClust	80.0	86.02	82.38	83.54	95.10	80.12	0:01.65
CD-HIT	80.0	92.08	89.55	87.30	100.00	85.77	0:00.32
UCLUST	80.0	71.88	58.05	47.75	100.00	60.21	0:00.27
MeShClust	85.0	80.28	79.30	76.24	96.03	71.98	0:23.90
CD-HIT	85.0	83.97	79.88	75.69	100.00	74.21	0:00.46
UCLUST	85.0	61.63	49.66	39.06	100.00	52.42	0:00.42
MeShClust	90.0	71.20	74.70	70.88	96.52	62.89	0:02.17
CD-HIT	90.0	69.38	64.13	56.51	100.00	59.17	0:01.03
UCLUST	90.0	45.84	31.26	22.05	100.00	43.70	0:00.52
MeShClust	95.0	65.79	69.62	60.01	98.74	57.79	0:02.02
CD-HIT	95.0	43.95	33.93	25.51	100.00	43.34	0:00.46
UCLUST	95.0	25.77	17.42	11.35	100.00	37.20	0:00.28
Hybrid clustering	—	98.35	96.44	95.06	100.00	96.88	0:09.20

[1] simulation data set 5 was obtained by DeepSimulator.

[2] Ten thousand sequences comprise this data set. Sequence lengths about 95 base pairs. The dataset has 20 classes. The performances were compared using these four identity scores: 80%, 85%, 90%, 95%.

Table S15: Comparison of the performances of the three tools and our method on the simulation data set 6

Tool	Identity(%)	AMI(%)	FMI(%)	ACC(%)	HOMO(%)	COMP(%)	Time(min:sec)
MeShClust	80.0	91.23	83.59	85.07	96.43	87.86	0:31.24
CD-HIT	80.0	93.46	88.53	84.54	100.00	88.71	0:00.32
UCLUST	80.0	81.74	71.49	62.43	100.00	74.10	0:00.27
MeShClust	85.0	88.09	83.86	80.94	98.70	82.21	0:01.95
CD-HIT	85.0	87.55	80.41	75.11	100.00	80.66	0:00.63
UCLUST	85.0	72.36	61.43	52.55	100.00	66.10	0:00.39
MeShClust	90.0	84.54	80.70	75.68	99.73	77.60	0:56.69
CD-HIT	90.0	74.70	64.18	55.83	100.00	67.92	0:00.82
UCLUST	90.0	55.54	41.41	32.04	100.00	56.19	0:00.51
MeShClust	95.0	72.33	69.29	60.97	99.24	66.86	0:02.43
CD-HIT	95.0	49.61	35.44	27.06	100.00	53.70	0:00.46
UCLUST	95.0	29.48	19.39	12.59	100.00	47.58	0:00.33
Hybrid clustering	—	97.38	94.55	91.55	99.97	95.17	0:08.53

[1] simulation data set 6 was obtained by DeepSimulator.

[2] Ten thousand sequences comprise this data set. Sequence lengths about 95 base pairs. The dataset has 50 classes. The performances were compared using these four identity scores: 80%, 85%, 90%, 95%.

S4 Pseudo code about hybrid clustering algorithm

The following 5 pseudo-codes describe the clustering process of the hybrid clustering algorithm in detail.

Algorithm 1 Get the initial clustering results.

Input: A set of t nucleotide sequences $N = \{n_1, n_2, ..., n_t\}$ sorted by decreasing length and *identity*

Output: Clusters of sequences and their respective centers

```
while N is not empty do
    Find the first sequence in N as center_now
    cluster_now ← {center_now}
    NS ← all sequences in N and be filtered by short word about the center_now
    S ← N − NS
    if S is not empty then
        for sequence x in S do
            Alignment with center_now to get similarity c
            if c ≥ identity then
                cluster_now ← x ∪ cluster_now
            end if
        end for
        Add center_now to Centers
        Add cluster_now to Clusters
        N ← N − cluster_now
    else
        Add center_now to Centers
        Add cluster_now to Clusters
        N ← N − cluster_now
    end if
end while
```

Algorithm 1 shows the main steps of the initial clustering algorithm. The idea of algorithm is consistent with that of CD-HIT algorithm. First, the t nucleotide sequences are given, which are stored in the set N. The sequences in N are sorted by length, with the long sequence always coming before the short one. Then, the longest sequence is selected as the representative sequence, where a short word filter is set for the representative sequence. The sequences filtered by short words are stored in the set NS, where S is the complement of NS. The sequence in S is aligned with the representative sequence to obtain the similarity score. If the score is not less than the given *identity*, then this sequence and the representative sequence belong to the same cluster. After the alignment, the cluster set $cluster_{now}$ of the representative sequence is obtained, $N = N - cluster_{now}$. The longest sequence in N is continuously selected as the representative sequence, and then the cluster set about the representative sequence is obtained until N is empty and the algorithm ends.

Algorithm 1 can be used to obtain some clusters, which need to be merged in order to obtain more accurate clustering results. According to the nucleotide sequence, we can obtain the corresponding nanopore signal, according to these signals, combined with the DTW algorithm can achieve cluster combination. We use algorithm 2 to calculate a DTW distance threshold. If the DTW distance of two sequences is less than this threshold, we consider the two sequences belong to the same class.

Algorithm 2 Get the merge threshold

Input: A set of t nucleotide sequences $N = \{n_1, n_2, ..., n_t\}$ sorted by decreasing length and nanopore signal set $S = \{s_1, s_2, ..., s_t\}$ corresponding to each nucleotide sequence a interger k

Output: A merge threshold

Run algorithm 1 use a very high *Identity* to get *InitialClusters*

Sort the clusters in *InitialClusters* by size (descending)

Pick the top one percent clusters and get their representative signals.

Calculate all DTW distances between these representative signals, and record the maximum value as M and the minimum value as m.

$threshold = (m + M)/k$

Algorithm 3 shows the acquisition process of consensus signal set. Firstly, we can obtain the initial clustering result through algorithm 1. According to the size of the obtained clustering set, we can divide all clusters into two categories, namely good cluster(*GoodCluster*) and bad cluster. For all *GoodCluster*, we merge them according to the following rules: for each *GoodCluster*, 3 sequences are selected as representatives, corresponding nanopore signals of these sequences are found, and DTW distance is calculated. If the DTW distance between the corresponding representative signals of the two clusters is less than the *threshold*, then the two clusters are merged. Finally, the merged *GoodCluster* can be obtained, denoted as $GoodCluster'$. For each cluster set in $GoodCluster'$, we can get their representative signals, and the set of these representative signals is called $ConSigSet$.

Algorithm 4 shows the overall process of hybrid clustering algorithm. At the beginning, we run Algorithm 2 and Algorithm 3 to get the $threshold$, $ConSigSet$ and $GoodCluster'$. The sequence not in $GoodCluster'$ is placed in the set OSS. If the DTW distance between the signal corresponding to the sequence in OSS and the signal in $ConSigSet$ is less than the *threshold*, then the sequence belongs to the cluster corresponding to the signal in $ConSigSet$. If OSS is still not an empty set, then a sequence in OSS is selected as the representative, and the corresponding nanopore signal is found, and the DTW distance between this signal and the corresponding signal of other sequences in OSS is calculated. If the distance is less than *threshold*, then these sequences are in the same class as the representative sequences, and so on until the OSS is empty set algorithm ends. When running Algorithm 1, many ultra-short nanopore sequences due to base-calling errors were removed in order to ensure the accuracy of subsequent consensus sequences. The purpose of Algorithm 5 is to find the nanopore signals corresponding to these ultra-short sequences and complete the clustering of these sequences by signal retrieval e.g. a signal is taken as a representative, and the DTW distance between it and other signals is calculated. If the distance is less than the $threshold$, then these signals belong to the same class as the representative signals.

S5 Usage of our method

To utilize our tool, users are required to provide the following data:

- Raw nanopore signals: These are the original signals obtained from nanopore sequencing.
- DNA sequences after sequencing: The DNA sequences obtained through the sequencing process.
- Barcode sequences: The specific sequences corresponding to the barcodes used in the experiment.
- Adapter sequence: The adapter sequence used in the sequencing process.

The workflow involves using specific scripts as described below:

Algorithm 3 Get some information about the initial clustering results

Input: A set of t nucleotide sequences $N = \{n_1, n_2, ..., n_t\}$ sorted by decreasing length and *identity*

Output: A initial classification signal set and good cluster about Initial clustering results

function GETCONSEQSIGNAL(DNAcluster)
  $ConsensusSignal$=representive signal of DNAcluster
  return $ConsensusSignal$
end function
function GETMERTHCFSIGNAL(N,$Identity$)
  Run $Algorithm$ 2 to get a merge $threshold$, sorted $InitialClusters$
  Pick the top one percent clusters in sorted $InitialClusters$ as $GoodClusterSet$
  for $GoodCluster$ in $GoodClusterSet$ **do**
    Randomly select three signals corresponding to sequences from every $GoodCluster$
    if The pairwise distance between the selected signals is less than the given $threshold$ **then**
      Merge these $GoodCluster$ to get $GoodCluster'$
    end if
  end for
  for $GoodCluster'$ **do**
    Run $GetConSeqSignal(GoodCluster')$ to get $csig_{now}$
    Add $csig_{now}$ to $ConSigSet$
  end for
  return $threshold$, $ConSigSet$, all $GoodCluster'$
end function

- Data preparation: The user employs the "mainDataPreparation.py" script to extract the barcode data. In this step, the user inputs all the aforementioned data, and the output includes pseudo-barcode signals(the barcode signals extracted by our method), pseudo-barcode sequences(the barcode sequences extracted by our method), and the standard barcode signals corresponding to the barcode sequences.
- Clustering: The user then uses the "mainHybridClustering.py" script for clustering. Here, the user needs to provide the pseudo-barcode signals and pseudo-barcode sequences as input. The output is a file containing the clustering results.
- Demultiplexing: Finally, the user applies the "mainDemultiplexByClusteringRes.py" script to convert the clustering results into the final demultiplexing output. This step requires the clustering result file, pseudo-barcode signals, and the standard nanopore signals as input. The output is a file containing the demultiplexing result.
- By following this workflow, users can effectively utilize our toolkit for their data analysis needs.

Our tools and more detailed usage information can be found at the following links: `https://github.com/junhaiqi/Hybrid_clustering.git`

Algorithm 4 An overview of the algorithm about our clustering method

Input: A set of t nucleotide sequences $N = \{n_1, n_2, ..., n_t\}$ sorted by decreasing length and nanopore signal set $S = \{s_1, s_2, ..., s_t\}$ corresponding to each nucleotide sequence

Output: Clusters of nucleotide sequences

Run *Algorithm* 2 and *Algorithm* 3 to get *threshold*, *ConSigSet* and $GoodClusterSet'$
Add all elements from $GoodClusterSet'$ to $Clusters_{now}$
$OSS \leftarrow$ The set of nucleotide sequences not in $GoodClusterSet'$
while OSS is not empty **do**
  $RS \leftarrow$ Randomly select the signal corresponding to a sequence from $GoodClusterSet'$
  if The DTW distance between the signal corresponding to the nucleotide sequence S_{now} and a signal in $ConSigSet$ is less than $threshold$ and the DTW distance between the signal corresponding to the nucleotide sequence S_{now} and RS is less than $threshold$ **then**
    Add S_{now} to $GoodClusterSet'$
    Remove S_{now} from OSS
  end if
end while
if OSS is not empty **then**
  for s in OSS **do**
    $G \leftarrow$The set of all such sequences in OSS: the DTW distance between their corresponding signal and s is less than a given $threshold$
    $OSS \leftarrow OSS - G$
    if G is nonempty **then**
      $Clusters_{now} \leftarrow Clusters_{now} \cup G$
    end if
  end for
end if
$Length \leftarrow$ The number of sequences included in the $Clusters_{now}$
if $Length$ is equal to t **then**
  The final clustering result is $Cluster_{now}$
else
  Run algorithmic 5 to get final clustering result
end if

Algorithm 5 An algorithm to guarantee the integrity of clustering results

Input: The current $clusters_{now}$, A set of t nucleotide sequences $N = \{n_1, n_2, ..., n_t\}$

Output: The final Clusters of nucleotide sequences

$NN \leftarrow$ The set of sequences not from $Cluster$ in $Clusters_{now}$
for nn in NN **do**
    Randomly select the signal ss corresponding to a sequence from $Cluster$ in $Clusters_{now}$
    if The DTW distance between ss and nn is less than the $Threshold$ **then**
        $Cluster \leftarrow Cluster \cup nn$
        $Length \leftarrow Length + 1$
        $NN \leftarrow NN - nn$
    end if
end for
if $Length$ is not equal to t **then**
    for $sequence$ from NN **do**
        $Cluster_{new} \leftarrow$ The new cluster only contains $sequence$
        $Clusters_{now} \leftarrow Clusters_{now} \cup Cluster_{new}$
    end for
end if
