# Supplementary material for: HycDemux: a hybrid unsupervised approach for accurate barcoded sample demultiplexing in nanopore sequencing
Source: Genome Biol. 2023 Oct 5;24:222. doi: 10.1186/s13059-023-03053-1 (PMC10552309; doi:10.1186/s13059-023-03053-1)
Supplement: Supplementary file 2 — Additional file 2. Review history. [file 13059_2023_3053_MOESM2_ESM.docx]

**1^st^ round**

**Reviewer 1**

The authors develop their method with the purpose of barcoded sample demultiplexing, but none of that is part of the analysis or results, and this is at best misleading. Moreso, the only valid comparison would be against the demultiplexing done by Guppy, the current standard tool for this purpose.

Throughout the manuscript it is unclear what the authors mean with 'short reads'. Are those genuinely short DNA molecules that are being sequenced, or does this indicate short parts of sequenced long reads (e.g. those parts corresponding to the barcode)?

It is unclear if this is a tool that users can start using in their work right away, or if further development or packaging has to be done.

Can the authors comment on the length of the barcodes used by ONT? As far as I know these are longer than what is used in short read sequencing, exactly to include some redundancy and take the lower accuracy into account. As such there is also no need for completely nucleotide-level accuracy when aiming to demultiplex samples. Barcodes are not created randomly, but are specifically selected to have a sufficiently large edit distance with other barcodes in the set. The barcode lengths in Table 1 are as such not representative of what is used by ONT.

The performance of the tool is described with respect to 1 GB of data. Foremost, it is unclear if this indicates gigabytes or gigabases, and how this corresponds to the expected data output of a MinION or PromethION sequencer. Under the assumption that these are gigabases then this would correspond to 12 hours for a PromethION run (150Gb).

The fixed region of the barcode was located and segmented for your method, but it is unclear if the same fragment was used for CD-HIT and the other tools under comparison, or if those tools were used on the full read.

The comparison uses AMI, FMI, ACC, HOMO and COMP for evaluation, but what this means is not defined. The authors also don't discuss that e.g. UCLUST is 70x faster than the hybrid clustering, which seems highly relevant. I also cannot imagine that the CD-HIT, Uclust and MeShClust were designed for this purpose and I am therefore not too surprised about their mediocre performance.

The evaluation of the real-world dataset uses barcodes of 40 base pairs, which is a mismatch with the simulation.

Figure 1 is unreadable and incomprehensible.

Page 3 line 62 mentions that 120947 barcode reads/signals were obtained, which is not clear to me, and also raises the question for how many reads no barcode signal was found.

Throughout the manuscript there are various parts that would benefit from language editing. Terminology like "base-called nucleic bases" is highly unusual, and could be replaced by "bases" or "nucleotides" without loss of information.

**Reviewer 2**

Strengthens and Contributions
In this paper, the authors propose an interesting hybrid clustering algorithm for clustering short sequences with accuracy exceeding current standard clustering tools. In addition, this hybrid clustering algorithm can be applied to the demultiplexing problem in multi-sample sequencing.
Weakness
There still remain some concerns that may need authors to explain:
Major concerns:
(1)It is mentioned in the paper that the initial clustering algorithm is a greedy algorithm that can guarantee high homogeneity. However, is this greedy algorithm efficient for shorter DNA sequences (UMI, ~10bp)? If there is no effect and the size of the obtained clusters is very small, then all the clusters are not of high quality. Will this seriously affect the subsequent cluster merging process? If it works, can the authors try to use a hybrid clustering algorithm for UMI clustering?
(2)As far as I know, ONT provides many barcode suites. What is the distribution of barcode lengths in these suites? In the case of a sequence length of 145bp, the author also conducted experiments. However, the length of the barcode should not exceed 100bp? In addition, guppy is specifically aimed at the demultiplexing of barcode. In order to study whether the hybrid clustering algorithm is suitable for demultiplexing, the author should do a comparative experiment with guppy.
(3)If the barcode suite is used to complete multi-sample sequencing, the barcode sequence is known, and to complete a sequence demultiplexing, only a partial sequence alignment is required. This seems to be simpler than clustering, can the author further elaborate on the advantages of hybrid clustering algorithms?
Minor concerns:
(1)It is suggested that the authors try to combine Table 2~Table 7, or present the results in a more vivid way.
(2)Figure 4 and Figure 5 are aimed at the same problem, and it is suggested that the authors combine these two figures into one figure.

**Authors’ response**

Reviewer 1

Comments to the Author

Q: The authors present a method for clustering nanopore sequencing reads. The approach is interesting, but the manuscript is not without shortcomings. Please find my detailed comments below.

Ans: Thank you for the positive feedback and constructive comments. We have followed all of them to further improve the quality of the paper. Please find the point-to- point response below to your comments.

Q: 1) The authors develop their method with the purpose of barcoded sample demultiplexing, but none of that is part of the analysis or results, and this is at best misleading. Moreso, the only valid comparison would be against the demultiplexing done by Guppy, the current standard tool for this purpose.

Ans: Thank you for your excellent question, which is greatly appreciated as it significantly contributes to the improvement of our work. Our work was submitted one and a half years ago. The primary focus of our research was on the screening of a standard set of barcodes for demultiplexing purposes. Through the utilization of clustering methods, we were able to identify and select successfully generated barcodes. We would like to thank you again for your valuable feedback, which helped us identify content errors and deficiencies in our work. In addition, we have developed a module based on a voting mechanism to efficiently transform clustering results into final demultiplexing results, which is added into the revised manuscript and the github.

Our demultiplexing algorithm has been compared and analyzed with Guppy using seven multi-sample sequencing datasets. Our analysis encompasses the demultiplexing accuracy of each individual barcode, employing two evaluation metrics: average accuracy and minimum accuracy. Now we explain the concepts of average accuracy and minimum accuracy using an example. Consider a scenario with 10 sequences labeled as read1,read2,...,read10. The correct barcode labels for these sequences are 1, 1, 1, 1, 1, 2, 2, 2, 2, 2. Here, the label “1” (or “2”) indicates that the sequence carries the 1st (or 2nd) barcode. We want to assess the accuracy rates for these two barcodes. Assuming that the barcode labels obtained by the demultiplexing algorithm for the 10 sequences are 1, 2, 1, 1, 2, 1, 1, 2, 1, 2, we can calculate the accuracy rates. The accuracy rate for the first barcode is 3/5, indicating that 3 out of 5 sequences labeled as barcode 1 are correct. Similarly, the accuracy rate for the second barcode is 2/5, as 2 out of 5 sequences labeled as barcode 2 are correct. In this case, average accuracy is calculated as (3/5 + 2/5) / 2 = 0.5, and minimum accuracy is min(3/5,2/5) = 0.4. It is important to note that when dealing with a large number of barcodes, it is possible to have a demultiplexing result with a high average accuracy but a low minimum accuracy. This means that the algorithm performs well on the majority of barcodes but may be ineffective for certain barcodes. Therefore, solely relying on the average accuracy might not provide a comprehensive evaluation of the demultiplexing effectiveness. By including the minimum accuracy, we can better assess the performance of the demultiplexing algorithm. The results of our experiments indicate that our demultiplexing algorithm achieves a accuracy comparable to that of Guppy when the dataset contains a small number of barcodes (less than 96).

However, as the number of barcodes increases (greater than 200), Guppy encounters difficulties in demultiplexing certain barcodes (minimum accuracy < 0.6), whereas our algorithm consistently maintains stable performance (minimum accuracy > 0.9). The performance difference shown above can be attributed to the fact that Guppy is specifically designed for the official nanopore barcode suite, which comprises a limited number of barcodes (less than 96). As a result, Guppy struggles to effectively demultiplex datasets containing a large number of barcodes. Additionally, errors from basecalling can also impact the performance of Guppy. However, our algorithm demultiplexes a sequence using information about its cluster, which facilitates the demultiplexing of certain outlier sequences.

We added several paragraphs in Section 2.2 to describe how our algorithm compares to Guppy:

“Previous studies have demonstrated that hybrid clustering algorithm can deliver clustering results with high homogeneity and completeness. In this context, we will elaborate on how our hybrid clustering algorithm, coupled with a voting mechanism- based demultiplexing module, can attain demultiplexing results with high accuracy. We compare our method with the state-of-the-art demultiplexing tool, Guppy, and provide experiment details below.

Simulated multi-sample sequencing data: We obtained whole genome sequences for 17 Enterotoxigenic Escherichia coli strains[37], 45 historical Shigella strains[38], and 67 Shiga Toxin producing Escherichia coli strains[39] to construct multi-sample sequencing datasets. We constructed multiple multi-sample sequencing libraries by randomly interrupting genome sequences based on the sequencing length distribution of Oxford Nanopore Sequencing Technology (ONT). Figure 5A illustrates the resulting DNA sequence after library construction. We then used all completed sequences as input for DeepSimulator to generate simulated data. We ultimately constructed a total of 7 multi-sample sequencing data sets, as summarized in Table 5. In these datasets, D1∼D3 integrate the official nanopore barcode. In order to evaluate the robustness of the demultiplexing method, we increased the number of barcodes and generated simulated multi-sample sequencing data (D4∼D7), these barcodes were randomly generated, and a certain edit distance was guaranteed (edit distance = 14.5 ± 1.8). The barcode consist of three primary components: upstream flanking region, variable region, and downstream flanking region(Figure 5A). While the barcode lengths vary among the D4∼D7, the variable regions remain consistent at 24nt in length. For the EXP-NBD104 kit(D1), both upstream flanking region and downstream flanking region are 8nt long, resulting in a total barcode length of 40nt. In the case of the SQK- 16S024 kit(D2), upstream flanking region spans 15nt, downstream flanking region covers 20nt, and the barcode itself is 59nt in length. Finally, the EXP-PBC096 kit(D3) features a upstream flanking region of 7nt, a downstream flanking region of 29nt, and an overall barcode length of 60nt. For barcodes in D4∼D7, both upstream flanking region and downstream flanking region are 8nt long, resulting in a total barcode length of 40nt.

Extract data for demultiplexing: We obtained the barcode sequences (signals) from the multi-sample sequencing dataset [Figure 5B]. However, as errors may occur during the extraction process, we refer to the barcode sequence (signal) as a pseudo-barcode sequence (signal). These extracted pseudo-barcode sequences (signals) are utilized in the hybrid clustering and demultiplexing that follow [Figure 5C].

Evaluation index: Our analysis encompasses the demultiplexing accuracy of each individual barcode, employing two evaluation metrics: average accuracy and minimum accuracy. Now we explain the concepts of average accuracy and minimum accuracy using an example. Consider a scenario with 10 sequences labeled as read1,read2,...,read10. The correct barcode labels for these sequences are 1, 1, 1, 1, 1, 2, 2, 2, 2, 2. Here, the label “1” (or “2”) indicates that the sequence carries the 1st (or 2nd) barcode. We want to assess the accuracy rates for these two barcodes. Assuming that the barcode labels obtained by the demultiplexing algorithm for the 10 sequences are 1, 2, 1, 1, 2, 1, 1, 2, 1, 2, we can calculate the accuracy rates. The accuracy rate for the first barcode is 3/5, indicating that 3 out of 5 sequences labelled as barcode 1 are correct. Similarly, the accuracy rate for the second barcode is 2/5, as 2 out of 5 sequences labeled as barcode 2 are correct. In this case, average accuracy is calculated as (3/5 + 2/5) / 2 = 0.5, and minimum accuracy is min(3/5,2/5) = 0.4. It is important to note that when dealing with a large number of barcodes, it is possible to have a demultiplexing result with a high average accuracy but a low minimum accuracy. This means that the algorithm performs well on the majority of barcodes but may be ineffective for certain barcodes. Therefore, solely relying on the average accuracy might not provide a comprehensive evaluation of the demultiplexing effectiveness. By including the minimum accuracy, we can better assess the performance of the demultiplexing algorithm.

We conducted extensive experiments on datasets D1-D7 to showcase the effectiveness of our demultiplexing method, and the main experimental results are presented in Table 6. As shown in Table 6, both our demultiplexing method and Guppy achieve nearly perfect accuracy on datasets D1-D3, which have a limited number of carefully designed ONT barcodes (not exceeding 96). As the number of barcodes increases in D4-D7, Guppy’s average accuracy remains around 0.95 but its minimum accuracy drops below 0.7, indicating that Guppy fails to demultiplex sequences associated with certain barcodes. In contrast, our demultiplexing method maintains a stable performance with a minimum accuracy above 0.9. The randomly selected barcodes in D4-D7 may contain basecalling errors, which could impede Guppy’s demultiplexing accuracy based on DNA sequences. Additionally, as the number of barcodes increases, distinguishing between some barcodes becomes increasingly challenging, thereby making demultiplexing more difficult. Our demultiplexing algorithm utilizes both DNA sequence and nanopore signal information to achieve highly homogeneous clustering results and avoid basecalling errors. The voting mechanism is used to obtain demultiplexing results, which prevents abnormal sequences from affecting the accuracy of demultiplexing.

Our demultiplexing method is more efficient than Guppy (single-thread mode) when all barcode sequences (signals) have been extracted. In our test environment, the extraction efficiency of barcode data is around ∼255 reads/s. Based on this estimate, the time required to complete the demultiplexing of D1 is 7+47=54s, which means that our method can complete the demultiplexing of 3.2G data in 1 minute.”

Q: 2) Throughout the manuscript it is unclear what the authors mean with ‘short reads’. Are those genuinely short DNA molecules that are being sequenced, or does this indicate short parts of sequenced long reads (e.g. those parts corresponding to the barcode)?

Ans: Thank you for pointing out the problem in our context, it will help us improve the legibility of the article. ‘Short read’ refers to a DNA sequence of no more than 100 nt in length, which is “short parts of sequenced long reads (e.g. those parts corresponding to the barcode)”.

We have explained this in the abstract by adding a comment:

“Comprehensive experiments demonstrate that our approach outperforms all the traditional unsupervised tools in short read(corresponds to a barcode within a longer sequence and has a length of less than 100 nucleotides) clustering, and performs more robustly than current state-of-the-art demultiplexing tool for complex multi-sample sequencing data”.

In Background (line 8):

“Usually, short nucleotide sequence corresponds to a barcode or special coded segment within a long read whose length is short than 100 nucleotides.”

Q: 3) It is unclear if this is a tool that users can start using in their work right away, or if further development or packaging has to be done.

Ans: Thank you for pointing this out. Over the course of a year and a half, we have made significant advancements and enhancements to our toolkit compared to the previous version. The key differences are as follows:

• Implementation in C++: The main functionalities of the new toolkit have been implemented in C++. Additionally, we have optimized the GPU module to leverage its full potential for calculating the Dynamic Time Warping (DTW) distance. As a result, the clustering efficiency has been further improved, achieving approximately 5 times faster performance.

• Addition of demultiplexing module: The new toolkit now includes a module specifically designed to convert clustering results into demultiplexed results. This enables users to obtain the final demultiplexing output conveniently. The optimized algorithm produced new experimental results, and all experimental results have been updated in Table 2.

In Supplementary Material S5, some paragraphs have been added to introduce the use of our tool:

“To utilize our tool, users are required to provide the following data:

• Raw nanopore signals: These are the original signals obtained from nanopore sequencing.

• DNA sequences after sequencing: The DNA sequences obtained through the sequencing process.

• Barcode sequences: The specific sequences corresponding to the barcodes used in the experiment.

• Adapter sequence: The adapter sequence used in the sequencing process. The workflow involves using specific scripts as described below:

• Data preparation: The user employs the “mainDataPreparation.py” script to extract the barcode data. In this step, the user inputs all the aforementioned data, and the output includes pseudo-barcode signals(the barcode signals extracted by our method), pseudo-barcode sequences(the barcode sequences extracted by our method), and the standard barcode signals corresponding to the barcode sequences.

• Clustering: The user then uses the “mainHybridClustering.py” script for clustering. Here, the user needs to provide the pseudo-barcode signals and pseudo-barcode sequences as input. The output is a file containing the clustering results.

• Demultiplexing: Finally, the user applies the “mainDemultiplexByClusteringRes.py” script to convert the clustering results into the final demultiplexing output. This step requires the clustering result file, pseudo-barcode signals, and the standard nanopore signals as input. The output is a file containing the demultiplexing result.

• By following this workflow, users can effectively utilize our toolkit for their data analysis needs.

Our tools and more detailed usage information can be found at the following links: https://github.com/ junhaiqi/Hybrid_clustering.git”

Q: 4) Can the authors comment on the length of the barcodes used by ONT? As far as I know these are longer than what is used in short read sequencing, exactly to include some redundancy and take the lower accuracy into account. As such there is also no need for completely nucleotide-level accuracy when aiming to demultiplex samples. Barcodes are not created randomly, but are specifically selected to have a sufficiently large edit distance with other barcodes in the set. The barcode lengths in Table 1 are as such not representative of what is used by ONT.

Ans: Thank you very much for pointing this out, we have noticed the issue. The focus of our work is to complete the accurate clustering of short sequences, which can be applied to the demultiplexing problem of ONT barcode. However, we ignore the specific length of the ONT barcode. In this regard, we have comprehensively investigated the relevant information of ONT barcode and supplemented the corresponding experiments. Thank you again for pointing out the deficiencies in our work. Currently, ONT mainly uses three barcode kits, namely EXP-NBD104, SQK- 16S024, and EXP-PBC096. The barcode in these kits consist of three primary components: upstream flanking region, variable region, and downstream flanking region(Figure 5 in main text). While the barcode lengths vary among the three kits, the variable regions remain consistent at 24nt in length.

For the EXP-NBD104 kit, both upstream flanking region and downstream flanking region are 8nt long, resulting in a total barcode length of 40nt. In the case of the SQK- 16S024 kit, upstream flanking region spans 15nt, downstream flanking region covers 20nt, and the barcode itself is 59nt in length. Finally, the EXP-PBC096 kit features a upstream flanking region of 7nt, a downstream flanking region of 29nt, and an overall barcode length of 60nt.

Although ONT has not disclosed the specific design details of their barcodes, we can confirm that the edit distance between barcodes within each suite is substantial. For all kits, the edit distance between barcodes exceeds 12.

The sequence lengths presented in Table 1 do not represent typical barcode lengths. However, the data generated from these sequences has provided initial validation of the viability of our hybrid clustering algorithm. Furthermore, it has shed light on the statistical relationship between sequence length and clustering performance. It is important to note that in the future, biologists may develop longer barcodes to expand the capacity of the barcode suite. In such cases, our algorithm holds potential for various applications. At the same time, We have utilized ONT’s barcode suite as well as sequences of the same length as the ONT barcodes to generate a total of seven datasets. These datasets were specifically created to evaluate the performance of our algorithm.

Q: 5) The performance of the tool is described with respect to 1 GB of data. Foremost, it is unclear if this indicates gigabytes or gigabases, and how this corresponds to the expected data output of a MinION or PromethION sequencer. Under the assumption that these are gigabases then this would correspond to 12 hours for a PromethION run (150Gb).

Ans: Thank you for pointing out the problem in our context. The “GB” used to describe the data size in the article refers to gigabytes, and all the data mentioned in the article are simulated nanopore sequencing data or from MinION sequencer.

In line 83 of the main text, we have clarified the meaning of “GB” to ensure accurate expression:

“Our algorithm incorporates a GPU-based parallel mechanism, which allows for the demultiplexing of 3.2GB(gigabytes) of data (nanopore signals + basecalled DNA sequences) in approximately 1 minute.”

For clarity, we have added sentences to the descriptive information in Table 5 (∼line 331):

“All datasets used to evaluate demultiplexing performance. “GB” is an abbreviation for gigabytes.”

Q: 6) The fixed region of the barcode was located and segmented for your method, but it is unclear if the same fragment was used for CD-HIT and the other tools under comparison, or if those tools were used on the full read.

Ans: Thank you for pointing out the problem in our context. All clustering tools and our algorithm use the same fragments, which means that CDHIT and other tools do not perform clustering on datasets consisting of full-length sequences

We have added the following sentences in Section 2.1.1 to emphasize this point:

“All the aforementioned datasets primarily consist of two main components. The first component comprises sequence fragments that represent the barcodes, while the second component consists of the nanopore signals that correspond to the barcode sequences.”

Q: 7) The comparison uses AMI, FMI, ACC, HOMO and COMP for evaluation, but what this means is not defined. The authors also don’t discuss that e.g. UCLUST is 70x faster than the hybrid clustering, which seems highly relevant. I also cannot imagine that the CD-HIT, Uclust and MeShClust were designed for this purpose and I am therefore not too surprised about their mediocre performance.

Ans: Thank you for pointing out the problem in our context. We have provided explanations for the meaning of these indicators in the descriptive information presented in Table 2. Additionally, the specific calculation process for these indicators is described in detail in Supplementary Material S1. UCLUST is capable of achieving fast clustering due to its utilization of the non- alignment technique. This technique allows for the identification of sequences that are dissimilar to the query sequence in advance, thereby reducing the number of comparisons required. However, this approach may mistakenly classify many similar sequences as dissimilar, leading to a larger number of clusters in the final clustering result.

On the other hand, our hybrid clustering algorithm continuously refines the initial clustering results. This refinement process involves calculating a significant number of DTW (Dynamic Time Warping) distances, which is the main factor affecting the efficiency of our algorithm. To address this challenge, we have optimized our algorithm. In Table 2, it can be observed that our hybrid clustering algorithm is currently able to complete the clustering process of dataset S1 within 11 seconds. Although it is approximately ten times slower than UCLUST, the clustering accuracy of our algorithm is significantly higher.

UCLUST, CDHIT, and MeShClust are originally designed for the clustering of DNA sequences, and they can also be applied to the demultiplexing problem of barcodes. However, our experiments have revealed that these tools struggle to accurately cluster short sequences, often yielding results with low integrity. For instance, when processing a dataset of 300,000 sequences (with 350 barcodes), these clustering tools may produce approximately 100,000 clusters. Such results do not guarantee efficiency when deriving the final demultiplexed result from these clusters. In contrast, our hybrid clustering algorithm can generate approximately 1,500 clusters, ensuring the efficiency and reliability of obtaining demultiplexed results from the clustering outcomes.

We have added the following sentences to the description information in Table 2 to explain the meaning of AMI, FMI, ACC, HOMO and COMP:

“Performance comparison of the different clustering methods on the dataset S1 ∼ S6. Here, Identity is the parameter of clustering tools, AMI is the abbreviation of Adjusted Mutual Information, FMI is the abbreviation of FowlkesMallows Index, ACC is the abbreviation of Accuracy, HOMO is the abbreviation of Homogeneity, and COMP is the abbreviation of Completeness.”

In Section 2.1.3, some sentences are added:

“Detailed information on all evaluation metrics can be found in the supplementary material S1.”

Q: 8) The evaluation of the real-world dataset uses barcodes of 40 base pairs, which is a mismatch with the simulation.

Ans: Thank you for pointing out the problem in our context. To address this issue, we strictly followed the ONT multi-sample sequencing process when constructing the dataset. The length of the barcodes carried by the sequences in these data sets is consistent with the length of the barcodes provided by ONT. Table 5 summarizes the details of all datasets. Using these datasets, we perform a thorough comparison of our algorithm and Guppy.

Q: 9) Figure 1 is unreadable and incomprehensible.

Ans: Thank you for pointing out the problem in our context. We acknowledge the existence of this problem, and in order to provide a more comprehensive understanding, we have provided a detailed description of Figure 1.

The following is a new description of Figure 1:

“An example of clustering 30 sequences using our hybrid clustering algorithm(A, B and C) and the subsequent demultiplexing mechanism based on the clustering results(D). (A) We perform initial clustering on 30 sequences, resulting in 6 clusters. If a cluster contains a number of sequences greater than the GoodIndex, it is considered a good cluster. In this case, clusters C1, C2, and C3 are considered good clusters. (B) We attempt to merge the good clusters by selecting k signals from each cluster. If the distance values in the corresponding k × k DTW distance matrix are all smaller than a given threshold, we merge two clusters. In this case, C1 and C2 are merged into a single cluster. (C) We attempt to add sequences that are not in good clusters to the existing good clusters after the cluster merging is completed. We select a representative sequence from each good cluster, and calculate the DTW distance between the representative sequence and the sequences not in the good cluster. If the distance value is less than a given threshold, we add the sequence to the corresponding good cluster. (D) We demultiplex the cluster by selecting k signals within the cluster and converting all known barcode sequences into standard nanopore signals based on the official 6-mer table of Oxford Nanopore Sequencing Company. We then calculate the DTW distance matrix between these k signals and the standard nanopore signals, and find the row label corresponding to the minimum value of each column to obtain a one-dimensional row label matrix. Finally, we compute the mode of the row-labeled matrix and use it as the final demultiplexed result.”

Q: 10) Page 3 line 62 mentions that 120947 barcode reads/signals were obtained, which is not clear to me, and also raises the question for how many reads no barcode signal was found.

Ans: Thank you for pointing out the problem in our context. In order to validate the effectiveness of our hybrid clustering algorithm, we have utilized real datasets.

Consequently, we have rigorously constructed the barcode labels for each sequence (signal). The original dataset comprised 130,000 sequences (signals), out of which we were able to ascertain the labels of 120,947 sequences (signals). This indicates that we were unable to identify the barcode signals for 9053 sequences (signals), resulting in their subsequent filtering from further analysis.

We have re-described the construction process of the real dataset in Section 2.1.1:

“The real-world dataset came from eight R9.4 flow cells and six R9.5 flow cells, all sequenced with the EXP-NBD103 barcoding kit. We conducted a random selection of 130,000 sequences from the dataset provided by[33] and proceeded to calculate the edit distance between the barcode area in each sequence and the standard barcode area. If the edit distance exceeded 10, we labeled the sequence as “fuzzy,” indicating uncertainty regarding the presence of barcodes in these particular sequences. Ultimately, we constructed barcode labels for 120,947 sequences, forming a dataset known as the amplicon library. In amplicon library, we use Edlib[35] to locate the fixed region of the barcode and segment the barcode read, and use Semi-Global Dynamic Time Warping[36] to extract the corresponding raw signal of these barcodes. ”

Q: 11) Throughout the manuscript there are various parts that would benefit from language editing. Terminology like “base-called nucleic bases” is highly unusual, and could be replaced by “bases” or “nucleotides” without loss of information.

Ans: Thank you for pointing out the problem in our context. We have found this inappropriate expression in the text, and have replaced “base-called nuclear bases” in line 58 of the text with “nucleotides”.

Here is the new sentence after the correction:

“Our approach utilizes both the base-called nucleic base information and the raw current signal, in which the nucleotides are used to generate initial clustering and representation sequences, while the raw signals are used for cluster merging and refinement (Figure (A, B and C) gives an example).”

Reviewer 2

Comments to the Author

Q: In this paper, the authors propose an interesting hybrid clustering algorithm for clustering short sequences with accuracy exceeding current standard clustering tools. In addition, this hybrid clustering algorithm can be applied to the demultiplexing problem in multi-sample sequencing.

Ans: Thank you for the positive feedback and constructive comments. We have followed all of them to further improve the quality of the paper. Please find the point-to- point response below to your comments.

Major:

Q: 1) It is mentioned in the paper that the initial clustering algorithm is a greedy algorithm that can guarantee high homogeneity. However, is this greedy algorithm efficient for shorter DNA sequences (UMI, 10bp)? If there is no effect and the size of the obtained clusters is very small, then all the clusters are not of high quality. Will this seriously affect the subsequent cluster merging process? If it works, can the authors try to use a hybrid clustering algorithm for UMI clustering?

Ans: Thank you so much for the excellent question. We can see from Table 2 that the

clustering accuracy of traditional clustering tools is negatively correlated with the

sequence length. This means that shorter sequences lead to lower clustering

accuracy, although they still maintain higher homogeneity. Our initial clustering

algorithm employs a greedy strategy similar to these clustering tools. We fully agree

with you that on datasets generated from shorter sequences, our initial clustering

algorithm may yield very small cluster sizes. However, this does not mean that all

clusters lack high quality.

We have improved the criteria for identifying high-quality clusters. The clusters obtained from the initial clusters are sorted in descending order according to their size. We consider the top 1% of clusters as high-quality clusters, thus ensuring that cluster merging is not affected. Our algorithm was initially developed to address the demultiplexing of ONT barcode, specifically focusing on improving the accuracy and resolution of sequencing data. However, we acknowledge that our algorithm has certain limitations when it comes to UMI clustering. UMI clustering is an independent and intricate problem that necessitates the use of specially tailored algorithms. While our algorithm excels in demultiplexing, it may lack certain functionalities or optimizations required for handling UMI information, thereby not delivering performance comparable to algorithms designed specifically for UMI clustering.

We appreciate your suggestion and understand your expectations. Consequently, we are open to exploring the design and enhancement of algorithms for UMI clustering.

We recognize the significance of this matter and believe it warrants dedicated research and resolution. We will explore relevant literature and existing UMI clustering algorithms, seeking ways to integrate them with our algorithm or exploring potential collaborations to tackle this problem. Thanks again for this excellent question.

Q: 2) As far as I know, ONT provides many barcode suites. What is the distribution of barcode lengths in these suites? In the case of a sequence length of 145bp, the author also conducted experiments. However, the length of the barcode should not exceed 100bp? In addition, guppy is specifically aimed at the demultiplexing of barcode. In order to study whether the hybrid clustering algorithm is suitable for demultiplexing, the author should do a comparative experiment with guppy.

Ans: Thank you very much for pointing those problems in our context, we have noticed those issues. Currently, ONT mainly uses three barcode kits, namely EXP-NBD104, SQK-16S024, and EXP-PBC096. The barcode in these kits consist of three primary components: upstream flanking region, variable region, and downstream flanking region(Figure 5 in main text). While the barcode lengths vary among the three kits, the variable regions remain consistent at 24nt in length.

For the EXP-NBD104 kit, both upstream flanking region and downstream flanking region are 8nt long, resulting in a total barcode length of 40nt. In the case of the SQK- 16S024 kit, upstream flanking region spans 15nt, downstream flanking region covers 20nt, and the barcode itself is 59nt in length. Finally, the EXP-PBC096 kit features a upstream flanking region of 7nt, a downstream flanking region of 29nt, and an overall barcode length of 60nt.

Corresponding experiments were performed for a sequence length of 145 bp and these can be found in Supplementary Material S2. The purpose of these experiments was to demonstrate that DTW can be used as a reliable indicator for measuring similarity between nanopore signals. However, it should be noted that this specific sequence length does not represent the typical length of a barcode. We fully agree with you that barcodes should not be longer than 100bp. According to the above information, we can know that the typical length of the ONT barcode is between 40 and 60bp.

We greatly appreciate your suggestion to compare our approach with Guppy, as it plays a crucial role in further improving our work. We compare with Guppy on seven datasets and provide a comprehensive analysis of the experimental results. Our analysis focuses on evaluating the demultiplexing accuracy of each individual barcode, utilizing two evaluation metrics: average accuracy and minimum accuracy. Now we explain the concepts of average accuracy and minimum accuracy using an example. Consider a scenario with 10 sequences labeled as read1,read2,...,read10. The correct barcode labels for these sequences are 1, 1, 1, 1, 1, 2, 2, 2, 2, 2. Here, the label “1” (or “2”) indicates that the sequence carries the 1st (or 2nd) barcode. We want to assess the accuracy rates for these two barcodes. Assuming that the barcode labels obtained by the demultiplexing algorithm for the 10 sequences are 1, 2, 1, 1, 2, 1, 1, 2, 1, 2, we can calculate the accuracy rates. The accuracy rate for the first barcode is 3/5, indicating that 3 out of 5 sequences labeled as barcode 1 are correct. Similarly, the accuracy rate for the second barcode is 2/5, as 2 out of 5 sequences labeled as barcode 2 are correct. In this case, average accuracy is calculated as (3/5 + 2/5) / 2 = 0.5, and minimum accuracy is min(3/5,2/5) = 0.4. It is important to note that when dealing with a large number of barcodes, it is possible to have a demultiplexing result with a high average accuracy but a low minimum accuracy. This means that the algorithm performs well on the majority of barcodes but may be ineffective for certain barcodes. Therefore, solely relying on the average accuracy might not provide a comprehensive evaluation of the demultiplexing effectiveness. By including the minimum accuracy, we can better assess the performance of the demultiplexing algorithm. Our experimental results demonstrate that our demultiplexing algorithm achieves a level of accuracy comparable to that of Guppy when the dataset contains a small number of barcodes (less than 96). However, as the number of barcodes increases (greater than 200), Guppy encounters challenges in demultiplexing certain barcodes, resulting in a minimum accuracy of less than 0.6. In contrast, our algorithm consistently maintains stable performance with a minimum accuracy exceeding 0.9. The performance difference highlighted above can be attributed to the fact that Guppy is primarily tailored for the official nanopore barcode suite, which encompasses a limited number of barcodes (less than 96). Consequently, Guppy encounters difficulties when confronted with datasets that contain a substantial number of barcodes. Furthermore, the performance of Guppy can be affected by errors stemming from basecalling. In contrast, our algorithm employs cluster-specific information to demultiplex sequences, enabling effective demultiplexing of outlier sequences.

We added several paragraphs in Section 2.2 to describe how our algorithm compares to Guppy:

“Previous studies have demonstrated that hybrid clustering algorithm can deliver clustering results with high homogeneity and completeness. In this context, we will elaborate on how our hybrid clustering algorithm, coupled with a voting mechanism- based demultiplexing module, can attain demultiplexing results with high accuracy. We compare our method with the state-of-the-art demultiplexing tool, Guppy, and provide experiment details below.

Simulated multi-sample sequencing data: We obtained whole genome sequences for 17 Enterotoxigenic Escherichia coli strains[37], 45 historical Shigella strains[38], and 67 Shiga Toxin producing Escherichia coli strains[39] to construct multi-sample sequencing datasets. We constructed multiple multi-sample sequencing libraries by randomly interrupting genome sequences based on the sequencing length distribution of Oxford Nanopore Sequencing Technology (ONT). Figure 5A illustrates the resulting DNA sequence after library construction. We then used all completed sequences as input for DeepSimulator to generate simulated data. We ultimately constructed a total of 7 multi-sample sequencing data sets, as summarized in Table 5. In these datasets, D1∼D3 integrate the official nanopore barcode. In order to evaluate the robustness of the demultiplexing method, we increased the number of barcodes and generated simulated multi-sample sequencing data (D4∼D7), these barcodes were randomly generated, and a certain edit distance was guaranteed (edit distance = 14.5 ± 1.8). The barcode consist of three primary components: upstream flanking region, variable region, and downstream flanking region(Figure 5A). While the barcode lengths vary among the D4∼D7, the variable regions remain consistent at 24nt in length. For the EXP-NBD104 kit(D1), both upstream flanking region and downstream flanking region are 8nt long, resulting in a total barcode length of 40nt. In the case of the SQK- 16S024 kit(D2), upstream flanking region spans 15nt, downstream flanking region covers 20nt, and the barcode itself is 59nt in length. Finally, the EXP-PBC096 kit(D3) features a upstream flanking region of 7nt, a downstream flanking region of 29nt, and an overall barcode length of 60nt. For barcodes in D4∼D7, both upstream flanking region and downstream flanking region are 8nt long, resulting in a total barcode length of 40nt.

Extract data for demultiplexing: We obtained the barcode sequences (signals) from the multi-sample sequencing dataset [Figure 5B]. However, as errors may occur during the extraction process, we refer to the barcode sequence (signal) as a pseudo-barcode sequence (signal). These extracted pseudo-barcode sequences (signals) are utilized in the hybrid clustering and demultiplexing that follow [Figure 5C]. Evaluation index: Our analysis encompasses the demultiplexing accuracy of each individual barcode, employing two evaluation metrics: average accuracy and minimum accuracy. Now we explain the concepts of average accuracy and minimum accuracy using an example. Consider a scenario with 10 sequences labeled as read1,read2,...,read10. The correct barcode labels for these sequences are 1, 1, 1, 1, 1, 2, 2, 2, 2, 2. Here, the label “1” (or “2”) indicates that the sequence carries the 1st (or 2nd) barcode. We want to assess the accuracy rates for these two barcodes. Assuming that the barcode labels obtained by the demultiplexing algorithm for the 10 sequences are 1, 2, 1, 1, 2, 1, 1, 2, 1, 2, we can calculate the accuracy rates. The accuracy rate for the first barcode is 3/5, indicating that 3 out of 5 sequences labeled as barcode 1 are correct. Similarly, the accuracy rate for the second barcode is 2/5, as 2 out of 5 sequences labeled as barcode 2 are correct. In this case, average accuracy is calculated as (3/5 + 2/5) / 2 = 0.5, and minimum accuracy is min(3/5,2/5) = 0.4. It is important to note that when dealing with a large number of barcodes, it is possible to have a demultiplexing result with a high average accuracy but a low minimum accuracy. This means that the algorithm performs well on the majority of barcodes but may be ineffective for certain barcodes. Therefore, solely relying on the average accuracy might not provide a comprehensive evaluation of the demultiplexing effectiveness. By including the minimum accuracy, we can better assess the performance of the demultiplexing algorithm.

We conducted extensive experiments on datasets D1-D7 to showcase the effectiveness of our demultiplexing method, and the main experimental results are presented in Table 6. As shown in Table 6, both our demultiplexing method and Guppy achieve nearly perfect accuracy on datasets D1-D3, which have a limited number of carefully designed ONT barcodes (not exceeding 96). As the number of barcodes increases in D4-D7, Guppy’s average accuracy remains around 0.95 but its minimum accuracy drops below 0.7, indicating that Guppy fails to demultiplex sequences associated with certain barcodes. In contrast, our demultiplexing method maintains a stable performance with a minimum accuracy above 0.9.

The randomly selected barcodes in D4-D7 may contain basecalling errors, which could impede Guppy’s demultiplexing accuracy based on DNA sequences. Additionally, as the number of barcodes increases, distinguishing between some barcodes becomes increasingly challenging, thereby making demultiplexing more difficult. Our demultiplexing algorithm utilizes both DNA sequence and nanopore signal information to achieve highly homogeneous clustering results and avoid basecalling errors. The voting mechanism is used to obtain demultiplexing results, which prevents abnormal

sequences from affecting the accuracy of demultiplexing.

Our demultiplexing method is more efficient than Guppy (single-thread mode) when all barcode sequences (signals) have been extracted. In our test environment, the extraction efficiency of barcode data is around ∼255 reads/s. Based on this estimate, the time required to complete the demultiplexing of D1 is 7+47=54s, which means that our method can complete the demultiplexing of 3.2G data in 1 minute.”

Q: 3) If the barcode suite is used to complete multi-sample sequencing, the barcode sequence is known, and to complete a sequence demultiplexing, only a partial sequence alignment is required. This seems to be simpler than clustering, can the author further elaborate on the advantages of hybrid clustering algorithms?

Ans: Thank you for your excellent question, which is of great significance to the improvement of our work. We fully agree with your point that demultiplexing a sequence can be achieved by aligning it with known barcode sequences. However, we believe that this approach is inefficient, especially when dealing with a large number of sequences and barcodes. Let’s consider an example where we need to demultiplex 100,000 sequences using 100 barcodes. Theoretically, to complete the demultiplexing process for these sequences, we would need to perform approximately 100,000 × 100 comparisons, resulting in a total of 10,000,000 comparisons. If we assume each alignment takes 0.01 seconds, it would take a total of 100,000 seconds to complete the demultiplexing, which is quite time-consuming.

In contrast, our hybrid clustering algorithm significantly reduces the number of alignments required. Here’s how it works:

• Initial Clustering: Our algorithm employs a greedy strategy in the initial clustering step to filter out a large number of unnecessary comparisons. This allows us to quickly obtain an initial clustering result.

• Representative Units: Once we have the initial clusters, we extract representative units from each cluster. These representative units play a crucial role in the merging of clusters. The number of Dynamic Time Warping (DTW) comparisons required to complete the merging based on these representative units is considerably smaller. Additionally, we leverage a GPU-based acceleration mechanism to expedite this process.

• Refinement Process: The clustering results undergo a refinement process where local clustering is performed on a very small number of sequences. Again, the GPU acceleration mechanism ensures the efficiency of this process. To complete the demultiplexing, we employ a voting mechanism based on the approach you mentioned. However, instead of demultiplexing individual sequences, we directly demultiplex entire clusters. This means that the demultiplexed result is the same for each sequence within a cluster. We select k representative sequences from each cluster and perform DTW alignment between these k sequences and all standard barcode sequences. This generates k demultiplexed results for each cluster. We then select the demultiplexed result with the highest frequency as the final demultiplexed result for that cluster.

Assume that our hybrid clustering algorithm clusters q sequences, resulting in n clusters. These sequences carry m standard barcodes. The final demultiplexing result obtained from the clusters requires a total of n×k ×m alignments, where all sequences are directly aligned with the standard barcode sequences to obtain demultiplexed results. This amounts to q × m alignments for the entire set of sequences. Through our analysis, we have determined that our hybrid clustering algorithm yields highly accurate results. The number of final clusters obtained aligns closely with the actual number of barcodes present. This implies that the product of n × k × m is significantly smaller than q × m. Additionally, our voting mechanism improves the robustness of demultiplexing by successfully handling barcodes containing more basecalling errors when they are correctly clustered.

We have made several additions to Section 2.1 to highlight the significance of our hybrid clustering algorithm for demultiplexing:

“We demultiplex sequences based on the results of hybrid clustering algorithm, which poses a performance challenge for clustering. Here, we mainly evaluate the performance of clustering from two aspects, one is homogeneity and the other is completeness. High homogeneity means that the sequences in each cluster obtained by clustering have the same barcode. However, there is an extreme case where each cluster contains only one sequence and the homogeneity is 100%. Therefore, the clustering results also need to be evaluated through completeness. High completeness means that there is a relatively small difference between the number of clusters obtained in the end and the actual number of barcodes. Starting from the clustering results, the demultiplexing results are deduced. The main advantage is that under the clustering results with high homogeneity, the demultiplexing result of a sequence are determined by some sequences in the cluster where it is located. This makes the result of demultiplexing more robust. On the other hand, we point out that clustering does not affect the efficiency of demultiplexing. Given n sequences, assuming that these n sequences carry m different barcodes, in theory, the final demultiplexing result can be obtained by completing n × m alignments. Clustering n sequences, assuming that n1 clusters are finally obtained, and then pick k representative elements in each cluster, and the k representative elements determine the result of demultiplexing, which requires n1×k×m alignments, when the clustering result has high completeness, n1 × k will be much smaller than n, which means that the efficiency of demultiplexing will be greatly improved. At the same time, clustering results with high homogeneity will lead to highly accurate demultiplexing results. We conducted experiments to demonstrate the hybrid clustering algorithm’s ability to produce high homogeneity and completeness results. The experimental process and analysis are presented in detail below.”

Minor:

Q: 1) It is suggested that the authors try to combine Table 2∼Table 7, or present the results in a more vivid way.

Ans: Thank you for pointing out the problem in our context. We have merged Table 2∼Table 7, and the new merged table is Table 2 in the revised article.

Q: 2) Figure 4 and Figure 5 are aimed at the same problem, and it is suggested that the authors combine these two figures into one figure.

Ans: Thank you for pointing out the problem in our context. We have combined Figure 4 and Figure 5 and obtained a new figure, which is Figure 3 in the revised article.

**2^nd^ round**

**Reviewer 1**

As a follow-up on my previous request for clarification regarding the meaning of "short reads", I don't consider the explanation sufficient, and it remains highly confusing. There is no reason to use incorrect terminology stubbornly, as short read sequencing is Illumina sequencing (or other competitors such as MGI), not just a "short part of a sequenced long read". The title remains problematic, as such, and I would strongly encourage you to omit "short read" from the manuscript and to rewrite it with a synonym instead (e.g., read fragment, short sequence fragment, barcode fragment) to avoid this confusion and to convey your message properly. Since your main focus is demultiplexing, I wonder if "A hybrid unsupervised approach for accurate barcoded sample demultiplexing in nanopore sequencing" is sufficient and more to the point.

The comparison with Guppy is highly appreciated, and I understand the reason behind using both average accuracy and minimum accuracy for comparison. Yet, I wonder if it makes more sense to write the average accuracy to the more commonly used "precision" metric or positive predictive value (fraction of correctly identified barcodes). Based on your example, those would be equivalent. This also indicates that your method (unlike Guppy's) makes a decision for every read, and there is no group of undetermined reads for which no barcode could be confidently identified. If you have a group of undetermined reads, you could also calculate the recall/sensitivity of your method. Your current evaluation metric entirely ignores the number of undermined reads. In addition, it is unclear if Guppy was used on a GPU or CPU.

I do not understand what the further applications would be of clustering, apart from barcode demultiplexing, and suggest to the authors they put their comparison with Guppy first, as I assume that to be the most relevant to the reader. One might even consider moving the comparison with clustering methods to the supplementary material or clearly indicate its significance beyond barcode demultiplexing. Clearly, a lot of work went into the results presented in Table 2, but its direct impact to the field and implications remain unclear.

I do not think it makes any sense to compare Guppy on barcodes it was not trained to detect, i.e., more than the 96 barcodes provided by ONT, since Guppy explicitly expects the "known" set of barcodes. Of course, it is advantageous that your method can demultiplex more barcodes. Something that has been getting traction in the last year is single-cell RNA sequencing using the 10X method and ONT sequencing, and I'd like to know how well your method would perform for this application. In this case, a very high number of cell barcodes and UMIs need to be recognized without the high edit distance from the standard ONT barcodes. A relevant tool for comparison would then be Blaze (https://github.com/shimlab/BLAZE). But that highly relevant evaluation makes more sense in a separate manuscript.

You mention: "Specifically, our experiments were conducted using datasets characterized by a relatively high sequencing error rate of approximately 15%.", which is much higher than the error rates that nanopore sequencing commonly achieves these days, which are in the order of 1-2% (depending on the pore version, duplex sequencing, and basecalling model).

Your GitHub link returns a 404, presumably because the repository is still Private and not publicly shared.

**Reviewer 2**

The authors have satisfied all my requests. Thank you and congratulations.

**Authors’ response**

Q: 1) As a follow-up on my previous request for clarification regarding the meaning of “short reads”, I don’t consider the explanation sufficient, and it remains highly confusing. There is no reason to use incorrect terminology stubbornly, as short read sequencing is Illumina sequencing (or other competitors such as MGI), not just a “short part of a sequenced long read”. The title remains problematic, as such, and I would strongly encourage you to omit “short read” from the manuscript and to rewrite it with a synonym instead (e.g., read fragment, short sequence fragment, barcode fragment) to avoid this confusion and to convey your message properly. Since your main focus is demultiplexing, I wonder if “A hybrid unsupervised approach for accurate barcoded sample demultiplexing in nanopore sequencing” is sufficient and more to the point.

Ans: Thank you very much for pointing out our lack of expression. We have thoroughly understood your comments and made the necessary revisions in the manuscript to accurately convey and describe our work. Following your suggestion, we have replaced the term “short reads” with “short sequence fragments” to ensure precise terminology.

Furthermore, we have given careful consideration to the title you provided and believe it effectively emphasizes the core focus of our research. As a result, we have replaced the original title with “A hybrid unsupervised approach for accurate barcoded sample demultiplexing in nanopore sequencing” to emphasize the key point of our work.

Q: 2) The comparison with Guppy is highly appreciated, and I understand the reason behind using both average accuracy and minimum accuracy for comparison. Yet, I wonder if it makes more sense to write the average accuracy to the more commonly used “precision” metric or positive predictive value (fraction of correctly identified barcodes). Based on your example, those would be equivalent. This also indicates that your method (unlike Guppy’s) makes a decision for every read, and there is no group of undetermined reads for which no barcode could be confidently identified. If you have a group of undetermined reads, you could also calculate the recall/sensitivity of your method. Your current evaluation metric entirely ignores the number of undermined reads. In addition, it is unclear if Guppy was used on a GPU or CPU.

Ans: Thank you very much for your valuable suggestions and the issues you have pointed out. We fully concur with your comment that, in our specific problem, the average accuracy can indeed be considered equivalent to precision. Our experimental findings have demonstrated that while the algorithm exhibits a high average accuracy (precision), there exists a notable disparity with the minimum accuracy, indicating that the algorithm may encounter challenges in demultiplexing certain samples.

In the context of multi-sample sequencing, post-demultiplexing, it becomes imperative to analyze each sample individually. Consequently, the demultiplexing accuracy of each individual sample significantly impacts downstream analyses. We further illustrate by Figure S1 in Supplementary Material S1 that while the average accuracy (precision) provides an overall performance measure across all samples, it may not fully reflect the demultiplexing performance on individual samples.

Our algorithm assigns a barcode label to each sequence, which is not good for detecting some outlier data, thank you very much for pointing this out. To address this, we have made significant improvements to our scheme, incorporating two key enhancements. Firstly, during the data preparation stage, our scheme now includes a mechanism to detect whether the adapter sequence has been successfully inserted into the sequence. This is accomplished by calculating the edit distance between the standard adapter sequence and the given sequence. If the edit distance exceeds 0.45 times the length of the standard adapter sequence, the sequence is marked as “unclassified.” Secondly, for a cluster that contains only one sequence, we calculate the minimum DTW distance between the pseudo-barcode signal corresponding to that sequence and all barcode signals. If the distance exceeds a well-designed threshold, the sequence is also labeled as “unclassified.”

To better evaluate the effectiveness of our method, we have introduced negative samples into the dataset. These negative samples consist of sequences that either lack barcodes or only contain incomplete barcodes (with a missing rate exceeding 0.6). Our past evaluation metrics may not have been sufficiently comprehensive. In order to provide a more thorough assessment of our algorithm’s performance, we have incorporated the Recall metric. Additionally, we have included a new category, “unclassified,” in Table 6 and Table 7 to indicate the number of undetermined reads. Thank you for pointing out issues with using Guppy. Subsequently, we conducted further investigations into Guppy and familiarized ourselves with its GPU version. It is worth noting that Guppy’s GPU version solely focuses on extracting the barcode region from the sequence using GPU processing. Consequently, all experiments were conducted using the GPU version of Guppy. The updated experimental results are reflected in Table 6 and Table 7.

We have added some sentences in Section 5.2 to highlight the improvements of our method during the data preparation phase:

“Assuming that the standard adapter sequence has a length of n, if the edit distance between the standard adapter sequence and the DNA sequence exceeds 0.45 times n (using local alignment), the barcode label of the sequence is deemed ambiguous and marked as “unclassified”.”

We have added some sentences in Section 5.6 to highlight the improvements of our method during the demultiplexing phase:

“Specifically, we identify sequences with ambiguous barcode labels using a straightforward and predetermined DTW distance threshold. The first cluster obtained from the clustering results is often of high quality, serving as a basis for generating a DTW distance threshold. Here is the refined description of the process. Firstly, 100 sequences are randomly selected from the cluster. Secondly, the DTW distance matrix is calculated for their corresponding pseudobarcode signals. Next, the average value of the matrix elements is computed and referred to as “mean”. Finally, a threshold is set to c times the mean, with a default value of c as 1.65. In the case of a cluster set containing only one sequence, the following criterion is applied: If the minimum DTW distance between the standard barcode signal and the pseudo-barcode signal corresponding to this sequence exceeds the threshold, the barcode label of the sequence is considered ambiguous and marked as “unclassified”.”

We have added Figure S1 to Supplementary Material S1 to further illustrate the importance of the minimum accuracy, here is the legend:

“The left and right plots show the distribution of each barcode’s demultiplexing accuracy, and there are a total of two hundred accuracy rates. The average accuracy of the left subfigure is 0.955, and the average accuracy of the right subfigure is 0.989. Some accuracy rates in the left subfigure are between 0.6 and 0.9, while all accuracy rates in the right subfigure are greater than 0.9.”

We have added some sentences to Supplementary Material S1 to further illustrate the importance of the minimum accuracy:

“Figure S1 shows the demultiplexing of the two algorithms. We can see two points from Figure S1. The first is that although the average accuracy of the first algorithm is high, the demultiplexing accuracy of many samples is very low, which implies that the algorithm cannot demultiplex some samples. The second is that although the average accuracy rates corresponding to the two algorithms are not much different, the demultiplexing accuracy rates of the second algorithm are all above 0.9, which clearly shows that the second algorithm has successfully demultiplexed all samples. This shows that the minimum accuracy can more accurately evaluate the performance of the demultiplexing algorithm.”

We have added some sentences in Section 2.1.1 to emphasize the use of negative samples:

“We used a total of 11 multi-sample sequencing datasets (Table 5) to evaluate our algorithm, all datasets were augmented with an additional 1000 sequences that either lacked or had incomplete barcode regions (with a missing ratio greater than 0.6). These sequences were classified as negative samples and their correct barcode label should be“unclassified”.”

We have added a few sentences in Section 2.1.1 to emphasize the new evaluation metric that were added:

“In addition, we utilize the recall rate as a measure of the model’s performance in correctly identifying positive samples. The formula for calculating the recall rate is:

Recall = TP / (TP + FN). Here, TP represents true positive examples (the number of samples correctly predicted as positive by the model), and FN represents false negative examples (the number of samples that are actually positive but are incorrectly predicted as negative by the model).”

In Table 6, we have explicitly indicated in the description that we utilized the GPU version of Guppy:

“Performance of our method and Guppy (GPU version) on D1-D7 with a 10% ∼ 15% sequencing error rate.”

In Table 7, we have explicitly indicated in the description that we utilized the GPU version of Guppy:

“Performance of our method and Guppy (GPU version) on DB1-DB4 with a 2% ∼ 5% sequencing error rate.”

We have added some sentences to Section 2.1.2 to redescribe the efficiency of our algorithm and Guppy:

“Compared to Guppy, our demultiplexing method is slightly less efficient in terms of speed but the running time remains at the same order of magnitude, due to the fact that our algorithm involves a lot of DTW distance calculations. However, it is important to note that our method still achieves a high level of demultiplexing efficiency.”

We have added some sentences in Section 2.1.2 to describe the experimental results on “unclassified”:

“Additionally, we observed that our algorithm exhibits fewer instances of “unclassified” labels compared to Guppy, and it aligns more closely with the ground truth value of 1000. This indicates that our demultiplexing algorithm excels at accurately assigning the correct barcode label to each sequence.”

We have added some sentences in Section 2.1.2 to describe the experimental results on “Recall”:

“In terms of recall, our algorithm outperforms Guppy by approximately 3%.”

“As shown in Table 7, with the improvement of sequencing error rate, both our method and Guppy showed improved demultiplexing accuracy, which is expected as sequencing error rates are generally inversely related to algorithm’s accuracy. In terms of average accuracy and recall, our algorithm demonstrated an advantage of approximately 2% over Guppy.”

Q: 3) I do not understand what the further applications would be of clustering, apart from barcode demultiplexing, and suggest to the authors they put their comparison with Guppy first, as I assume that to be the most relevant to the reader. One might even consider moving the comparison with clustering methods to the supplementary material or clearly indicate its significance beyond barcode demultiplexing. Clearly, a lot of work went into the results presented in Table 2, but its direct impact to the field and implications remain unclear.

Ans: Thank you for pointing this out. We appreciate your suggestion to move the clustering-related content to the supplementary material. However, the editor has recommended that we retain the relevant results of clustering in the main text. We may not have adequately emphasized the importance of our clustering algorithm, and we would like to explain why we chose to include this section in the main text and describe it first.

Hybrid clustering plays a crucial role in our demultiplexing method, and it is essential to understand its significance. One major advantage of clustering is that, under clusters with high homogeneity, the demultiplexing result of a sequence is determined by other sequences within the same cluster, enhancing the robustness of the demultiplexing outcome.

Moreover, the accuracy of hybrid clustering directly influences the accuracy of subsequent demultiplexing, making it necessary to evaluate the performance of our clustering algorithm. We carefully considered the type of data on which we are performing clustering, i.e., nanopore signals or basecalled DNA sequences. Through experiments, we discovered that clustering directly based on nanopore signals achieves high accuracy but is inefficient, while clustering directly based on DNA sequences is efficient but lacks accuracy guarantees. These observations led us to design our hybrid clustering algorithm, which needs to be thoroughly evaluated.

Specificially, the utilization of GPU for distance computation and clustering has been crucial, as evidenced by our experiments. By harnessing the power of GPUs, we have effectively applied certain algorithms that are slow but accurate, such as DTW distance computation with a complexity of n2, to big data analysis. This has ensured both the accuracy and efficiency of the analysis process, and provide a reference for other work.

On the other hand, in order to maintain the logical flow of the manuscript, we have chosen to initially present the performance evaluation of our hybrid clustering algorithm before delving into the evaluation of the demultiplexing algorithm. The experiments presented in Table 2 demonstrate two key findings. Firstly, our hybrid clustering algorithm produces highly accurate clustering results, ensuring the subsequent demultiplexing process. Secondly, it exhibits an important characteristic:

the greedy strategy can rapidly generate clusters with high homogeneity. This feature highlights the fundamental concept of clustering: employing the well-established greedy strategy to swiftly obtain highly homogeneous clusters and subsequently exploring suitable merging strategies. Compared to clustering the original sequence set directly, this approach significantly reduces the complexity of clustering based on selected clusters. Notably, our hybrid clustering algorithm merges these clusters based on signal information, and we believe that there are more suitable cluster merging schemes for other clustering problems. This concept may also be applicable to the issue you mentioned regarding barcode identification in single-cell RNA-sequencing data. We can extract pseudo-barcodes from single-cell RNA-sequencing data, perform rapid clustering on them, and design an appropriate merging strategy to accomplish the final barcode identification.

Therefore, due to the significance and relevance of clustering in our demultiplexing method, we believe it is essential to retain the relevant content in the main text. If you still insist that it is important to first describe the results of demultiplexing, we will make the necessary changes, but this change may result in an incoherent logical flow.

Thanks again for pointing out this valuable problem.

We have added some sentences in Section 2 to emphasize the reason for describing the hybrid clustering algorithm first:

“We derive the results of demultiplexing by the results of hybrid clustering, implying that the results of clustering directly affect the results of demultiplexing. In this section, we first evaluate the performance of the hybrid clustering algorithm, and the experimental results show that it can generate high-quality clusters, which provides a strong guarantee for subsequent demultiplexing. Afterwards, we show that a voting-based demultiplexing module can derive demultiplexed results with high accuracy from clustered results.”

In Section 3 of the main text, we added some paragraphs to highlight the importance of our hybrid clustering algorithm:

“We perform demultiplexing based on the clustering results, which offers a significant advantage. Clustering, particularly in clusters with high homogeneity, determines the demultiplexing outcome of a sequence based on other sequences within the same cluster. This characteristic enhances the robustness of the demultiplexing process, as it ensures that sequences within a cluster contribute to the determination of the demultiplexed result.”

“Additionally, we have integrated a GPU-based module into our algorithm, specifically designed for computing the DTW distance matrix between time series. This module proves highly efficency of GPU powered clustering, when dealing with time series datasets. The utilization of GPU for distance computation and clustering has been crucial, as evidenced by our experiments. By harnessing the power of GPUs, we have effectively applied certain algorithms that are slow but accurate, such as DTW distance computation with a complexity of n2, to big data analysis. This has ensured both the accuracy and efficiency of the analysis process, and has the potential to inspire future work in this area.

Through extensive experiments, we made an interesting observation regarding clustering tools and their clustering accuracy. While some clustering tools may not achieve high clustering accuracy, we found that certain tools utilizing greedy strategies, such as CDHIT, can ensure near-perfect homogeneity. This discovery has led to the emergence of a new clustering concept: employing a greedy strategy to rapidly obtain highly homogeneous clusters, and subsequently merging these clusters in a careful manner to continually improve clustering accuracy. By employing a suitable merging strategy for these initial clusters, we can achieve clustering results with significantly higher accuracy. Additionally, the complexity of clustering is substantially reduced when starting from these initial clusters, as compared to the original sequence set. This strategy can be seamlessly applied to DNA sequence clustering problems once an appropriate cluster merging scheme is established.”

Q: 4) I do not think it makes any sense to compare Guppy on barcodes it was not trained to detect, i.e., more than the 96 barcodes provided by ONT, since Guppy explicitly expects the “known” set of barcodes. Of course, it is advantageous that your method can demultiplex more barcodes. Something that has been getting traction in the last year is single-cell RNA sequencing using the 10X method and ONT sequencing, and I’d like to know how well your method would perform for this application. In this case, a very high number of cell barcodes and UMIs need to be recognized without the high edit distance from the standard ONT barcodes. A relevant tool for comparison would then be Blaze (https://github.com/shimlab/BLAZE). But that highly relevant evaluation makes more sense in a separate manuscript.

Ans: Thank you for your valuable suggestions, which provide guidance for our future work. Regarding the design details of ONT barcodes, we are aware that the official specifications have not been announced. Therefore, we may not be able to determine whether the barcode classification is done by trained machine learning methods or just edit distance comparision. Based on the existing barcode collection, we can infer that ONT barcodes maintain a relatively large edit distance (Not less than 12). In fact, Guppy’s usage documentation provides clear instructions for the demultiplexing process of non-ONT barcode sets, indicating that some users may not utilize ONT barcodes in their experiments. Therefore, Guppy is more likely to utilize sequence alignment technology instead of machine learning methods that necessitate data training for barcode demultiplexing. Additionally, other studies imply the usage of a large number of non-ONT barcodes. For example, Joaqu´ın Ezpeleta et al designed “NS-watermark barcodes” and combined them with nanopore sequencing for corresponding biological experiments (https://www.nature.com/ articles/s41598-022- 11656-0). Srivathsan et al selected the COI gene in the mitochondria of biological cells as barcodes, which were applied to nanopore sequencing (https://bmcbiol.biomedcentral.com/articles/10.1186/s12915-021-01141-x).

We are grateful for pointing out the relevant issues in single-cell RNA sequencing. We have conducted related research in this area, and it is likely to be the focus of our future work. In particular, we have extensively studied Blaze and analyzed relevant software. Barcode identification in single-cell RNA sequencing data is a critical challenge. The barcodes in the data are derived from a “white list” containing millions of barcodes. Unlike the multi-sample sequencing problem, the set of barcodes in RNA- Seq data is uncertain. We only know that they come from the “white list” and have an approximate number, making it a more complex task. However, this issue has a strong connection to our algorithm. If our algorithm is applied to this problem, two challenges must be addressed. The first is how to accurately extract the corresponding barcode sequence (signal) region, and the second is how to efficiently identify barcodes based on the clustering results. Once the first problem is solved, a natural approach would be to determine the consensus sequence based on the clusters in the clustering results and assign the barcode label accordingly. To ensure confidence, it may be necessary to consider clusters with a certain number of sequences. Solving these problems may require significant modifications to our scheme to better suit the barcode identification problem in single-cell RNA sequencing data. We appreciate your excellent comments, and we have provided corresponding discussions in the “Discussion” to clarify the focus of our future work.

To emphasize the significance of conducting experiments on datasets containing a large number of non-ONT barcodes, we added some sentences to Section 2.1.1:

“In order to evaluate the robustness of the demultiplexing method and the performance of demultiplexing a large number of non-ONT barcodes [44,45].”

To emphasize the focus of our future work, we have added some additional sentences to Section 3:

“Furthermore, barcoding is not only applicable to the multi-sample sequencing but also finds significant utility in the realm of single-cell RNA sequencing. By employing the 10X method in conjunction with ONT sequencing, RNA isoforms can be quantified at the individual cell level. The combination of ONT sequencing and the 10X method generates vast amounts of data, encompassing thousands of barcodes. These barcodes originate from a “white list” consisting of millions of barcodes. In downstream analysis, accurately identifying the barcodes within the sequences is the crucial initial step, as sequences with the same barcode are presumed to originate from the same cell. In response to this specific challenge, we aim to develop a more adaptive algorithm building upon our current work.”

Q: 5) You mention: “Specifically, our experiments were conducted using datasets characterized by a relatively high sequencing error rate of approximately 15%.”, which is much higher than the error rates that nanopore sequencing commonly achieves these days, which are in the order of 1-2% (depending on the pore version, duplex sequencing, and basecalling model).

Ans: Thank you very much for pointing this out, we have noticed the issue. When we submitted this work (a year and a half ago), ONT had a high sequencing error rate, and the accepted sequencing error rate was ∼ 15%[1], so our experiments were all done on datasets containing high sequencing error rates. We have noticed an improvement in sequencing accuracy for ONT[2,3], and performed experiments on datasets containing more realistic sequencing error rates (2% ∼ 5%). Although ONT has reported high sequencing accuracy, the sequencing accuracy on non-model organisms is not easily predictable, and S. Ferguson also clearly pointed out that the sequencing accuracy reflected in the real project is lower than that reported by ONT[4]. Additionally, it is worth mentioning that ONT Direct RNA sequencing still produces data with a relative high error rate[5,6]. Considering these factors, conducting experiments on datasets with high sequencing error rates still holds significance, and therefore, we have retained the relevant content in the main text.

Experimental results on datasets with more realistic sequencing error rates are shown in Table 7. From Table 7, we can see that the demultiplexing accuracy of our algorithm and Guppy has improved, because the datasets in Table 7 has a lower sequencing error rate. In addition, Guppy still faces challenges in accurately separating certain samples, resulting in a minimum accuracy below 0.8. In contrast, our demultiplexing method outperforms Guppy, achieving approximately 2% higher average accuracy and recall for demultiplexing, as well as approximately 15% higher minimum accuracy.

[1] Wang, Y., Zhao, Y., Bollas, A., Wang, Y., Au, K.F.: Nanopore sequencing technology, bioinformatics and applications. Nature biotechnology 39(11), 1348–1365 (2021). https://www.nature.com/articles/ s41587-021-01108-x#citeas

[2] Sereika, M., Kirkegaard, R.H., Karst, S.M., Michaelsen, T.Y., Sørensen, E.A., Wollenberg, R.D., Albertsen, M.: Oxford nanopore r10. 4 long-read sequencing enables the generation of near-finished bacterial genomes from pure cultures and metagenomes without short-read or reference polishing. Nature methods 19(7), 823–826795 (2022). https://www.nature.com/articles/s41592-022-01539-7

[3] Sanderson, N.D., Kapel, N., Rodger, G., Webster, H., Lipworth, S., Street, T.L., Peto, T., Crook, D., Stoesser, N.: Comparison of r9. 4.1/kit10 and r10/kit12 oxford nanopore flowcells and chemistries in bacterial genome reconstruction. Microbial genomics 9(1) (2023). https://www.ncbi.nlm.nih.gov/pmc/articles/PMC9973852/

[4] Ferguson, S., McLay, T., Andrew, R.L., Bruhl, J.J., Schwessinger, B., Borevitz, J., Jones, A.: Species-specific800 basecallers improve actual accuracy of nanopore sequencing in plants. Plant Methods 18(1), 1–11 (2022). https: //plantmethods.biomedcentral.com/articles/10.1186/s13007-022-00971-2

[5] Jain, M., Abu-Shumays, R., Olsen, H.E., Akeson, M.: Advances in nanopore direct rna sequencing. Nature Methods 19(10), 1160–1164 (2022). https://www.nature.com/articles/s41592-022-01633-w

[6] Liu-Wei, W., van der Toorn, W., Bohn, P., Holzer, M., Smyth, R., von Kleist, M.: Sequencing accuracy and¨ systematic errors of nanopore direct RNA sequencing. bioRxiv, 2023–03 (2023). https://www.biorxiv.org/ content/10.1101/2023.03.29.534691v1.full.pdf

We have added some sentences in Section 2.1.2 to describe the comparison between our algorithm and Guppy on Recall:

“In terms of recall, our algorithm outperforms Guppy by approximately 3%.”

We have added some sentences to describe how our algorithm compares with Guppy on all new datasets with a more realistic error rate, in Section 1:

“Additionally, our demultiplexing algorithm outperforms the state-of-the-art method by approximately 15% on datasets with sequencing errors 2% ∼ 5%.”

In Section 2.1.2:

“As shown in Table 7, with the improvement of sequencing error rate, both our method and Guppy showed improved demultiplexing accuracy, which is expected as sequencing error rates are generally inversely related to algorithm’s accuracy. In terms of average accuracy and recall, our algorithm demonstrated an advantage of approximately 2% over Guppy. In addition, it is worth noting that even with these improvements, the minimum accuracy of Guppy remains below 0.8, and our algorithm outperforms Guppy by ∼ 15%. This shows that under the current state-of-the-art sequencing accuracy, Guppy still cannot successfully demultiplex some samples, while our algorithm guarantees a demultiplexing accuracy above 0.9.”

In Section 3, we added some sentences to emphasize our future work:

“In recent years, significant advancements have been made in ONT Direct RNA sequencing. This approach eliminates the need for reverse transcription of RNA into cDNA, thereby mitigating potential issues associated with introducing errors or losing information during transcription. However, it is important to note that individual sequencing of RNA molecules often yields data with a relatively high error rate[46,47]. On the other hand, the combination of RNA molecules and barcodes also enables multi-sample sequencing[34]. Through experiments, we can see that our demultiplexing algorithm can successfully complete the demultiplexing of multiple samples on datasets with a high error rate, which implies that our algorithm can be applied to the demultiplexing of RNA samples. This is also the focus of our future work.”

Q: 6) Your GitHub link returns a 404, presumably because the repository is still Private and not publicly shared.

Ans: Thank you very much for pointing this out. We carefully checked the permissions on our repository to make sure it was publicly available. You can try to visit https://github.com/junhaiqi/Hybrid_clustering.git again with Google Chrome. If you still can’t get it, you can try to visit the link:

https://pan.baidu.com/s/1kCsuTVpv0s4ihF8hnpE6Fg?pwd=hycl to get the compressed package about it.

**3^rd^ round**

**Reviewer 1**

I wish to congratulate the authors with their work and excellent paper, and very much appreciate their response to my suggestions. I have no further concerns.
